# Engineered Half-Unit-Cell MoS_2_/ZnIn_2_S_4_ Monolayer Photocatalysts and Adsorbed Hydroxyl Radicals-Assisted Activation of C_α_–H Bond for Efficient C_β_–O Bond Cleavage in Lignin to Aromatic Monomers

**DOI:** 10.1021/acsami.4c10515

**Published:** 2024-08-31

**Authors:** Zongyang Yue, Guanchu Lu, Wenjing Wei, Yi Huang, Zheng Chen, Fergus Dingwall, Shibo Shao, Xianfeng Fan

**Affiliations:** † Institute for Materials and Processes, School of Engineering, The University of Edinburgh, Edinburgh EH9 3BF, U.K.; ‡ Petrochemical Research Institute, PetroChina Company Limited, Beijing 102206, China

**Keywords:** half-unit-cell MoS_2_/ZnIn_2_S_4_ monolayer, hydrogen transfer efficiency, activation of C_α_−H bond, C_β_−O bond cleavage, lignin valorization

## Abstract

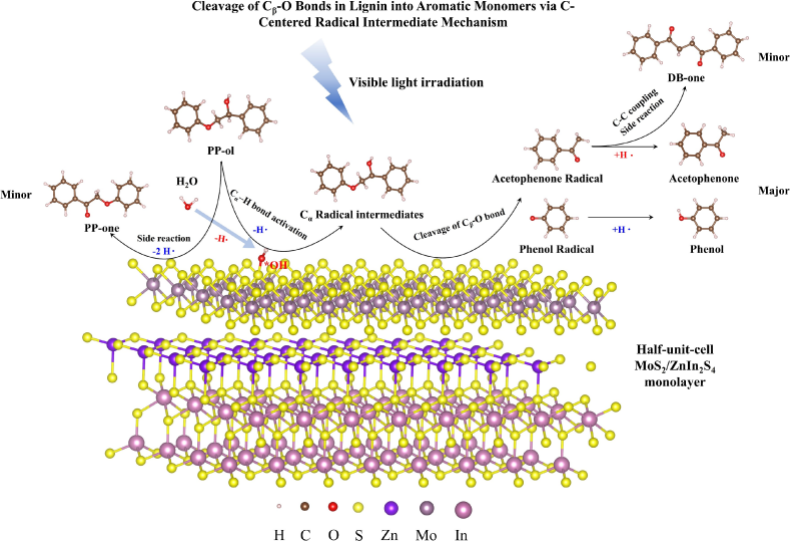

Photocatalysis has high potential in the cleavage of C_β_–O bond in lignin into high-value aromatic monomers; however, the inefficient C_α_–H bond activation in lignin and a low hydrogen transfer efficiency on the photocatalyst’s surfaces have limited its application in photocatalytic lignin conversion. This study indicates that the cleavage of the C_β_–O bond can be improved by the generation of the C_α_ radical intermediate through C_α_–H bond activation, and the formation of desirable aromatic products can be significantly improved by the enhanced hydrogen transfer efficiency from photocatalyst surfaces to aromatic monomeric radicals. We elaborately designed the half-unit-cell MoS_2_/ZnIn_2_S_4_ monolayer with a thickness of ∼1.7 nm to promote the hydrogen transfer efficiency on the photocatalyst surfaces. The ultrathin structure can shorten the diffusion distance of charge carriers from the interior to the surfaces and tight interface between MoS_2_ and ZnIn_2_S_4_ to facilitate the migration of photogenerated electrons from ZnIn_2_S_4_ to MoS_2_, therefore improving the selectivity of desirable products. The adsorbed hydroxyl radical (*OH) on the surfaces of MoS_2_/ZnIn_2_S_4_ from water oxidation can significantly reduce the bond dissociation energy (BDE) of C_α_–H bond in PP-ol from 2.38 to 1.87 eV, therefore improving the C_α_–H bond activation. The isotopic experiments of H_2_O/D_2_O indicate that the efficiency of *OH generation is an important step in C_α_–H bond activation for PP-ol conversion to aromatic monomers. In summary, PP-ol can completely convert to 86.6% phenol and 82.3% acetophenone after 1 h of visible light irradiation by using 3% MoS_2_/ZnIn_2_S_4_ and the assistance of *OH, which shows the highest conversion rate compared to previous works.

## 1 Introduction

Lignocellulosic biomass consists of cellulose (∼40%–60%), hemicellulose (∼20%–40%), and lignin (∼10%–24%), in which cellulose and hemicellulose have been used to produce microfibrils and C_5_/C_6_ chemicals, respectively; however, lignin is still barely utilized due to its structural complexity and recalcitrance.^1^ Lignin is an aromatic biopolymer composed of aromatic monomers with strong C_β_–O and C–C linkages. The aromatic monomers are platform chemicals for perfume industry, pharmaceuticals, and agriculture and could be produced from lignin through the cleavage of C_β_–O bond in lignin, as more than 50% of aromatic units in lignin are connected with this type of bond.^2−4^ Therefore, the catalytic cleavage of the C_β_–O bond in lignin is the critical step for effectively converting lignin to high-value aromatic products. Traditional thermo-catalytic strategies have been developed in the cleavage of C_β_–O bond to aromatic monomeric products, such as methylcyclohexane, cyclohexanol and benzene, but the resulting products are generally low-functionalized and low-value aromatic hydrocarbons.^5−10^ In addition, traditional thermocatalytic methods are typically conducted under temperatures ranging from 200 to 300 °C, and high-pressure H_2_ within the range of 0.5–0.7 MPa, therefore increasing the energy consumption and production cost.^10−12^

**Scheme 1:**
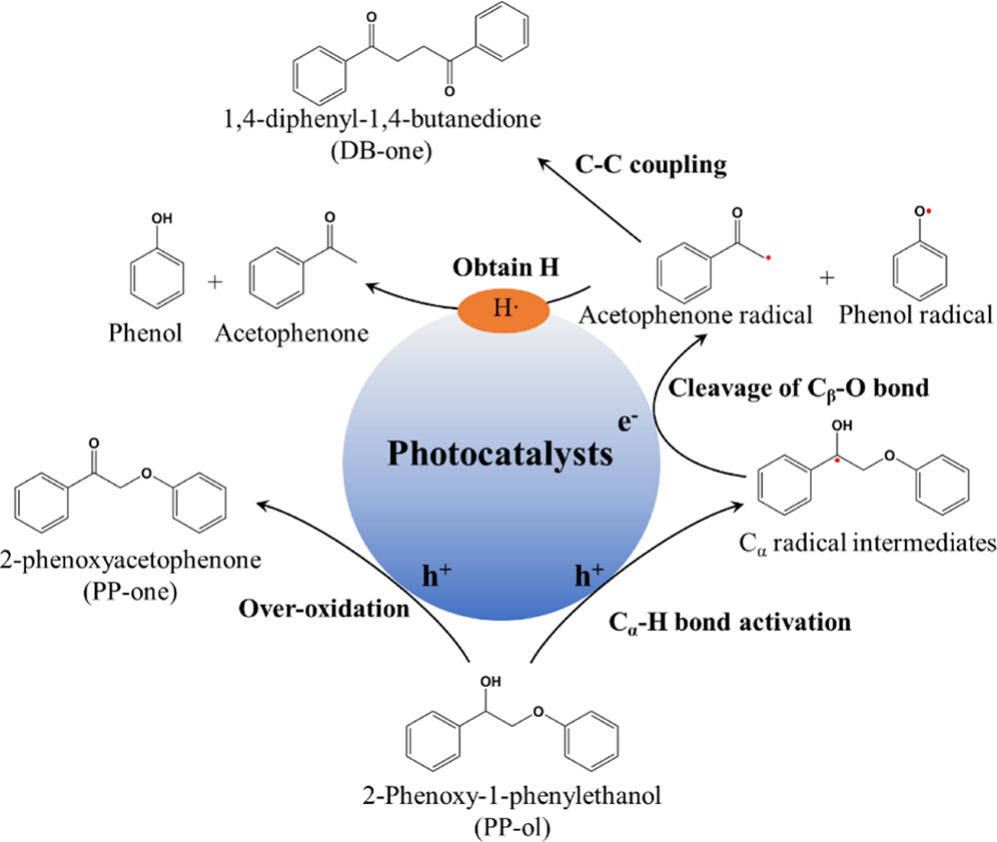
C_α_ Radical Intermediate Mechanism of C_β_–O Bond Fragmentation in PP-ol over Reported Photocatalysts Reproduced from refs (3,13),^15^ Copyright [2017, 2019, and 2020] American Chemical Society.

Photocatalysis has the promising potential for effectively converting lignin into aromatic monomers with highly functionalized hydrocarbons under the mild conditions.^2,3,13−17^ However, the reaction rate and its selectivity for photocatalytic cleavage of the C_β_–O bond to aromatic monomeric products need to be further improved. In the lignin fragmentation process, C_α_–H bond activation in the generation of C_α_ radical intermediates and hydrogen transfer efficiency in the formation of aromatic monomers are critical steps in the cleavage of C_β_–O bonds to aromatic monomers. Previous works have widely used PP-ol as the lignin model compound for cleaving the C_β_–O bonds to acetophenone and phenol as the target aromatic monomers, due to its structural similarity to the lignin compounds with C_β_–O bonds.^2,3,13−15^ In detail, as shown in Scheme 1, the C_α_ radical intermediates are initially generated through the C_α_–H bond activation in PP-ol by photogenerated holes and then the C_β_–O bond cleavage to form acetophenone radicals and phenol radicals through photoexcited electrons. Both types of radicals can obtain hydrogen from the surface of photocatalysts, resulting in the formation of acetophenone and phenol as target aromatic monomers.^18^ It is worth noting that the formation of C_α_ radical intermediate from lignin through the C_α_–H bond activation can enhance the cleavage rate of C_β_–O bonds in lignin, as the C_α_ radical intermediates can significantly decrease the BDE of C_β_–O bonds from 55 kcal mol^–1^ in PP-ol to 7.8 kcal mol^–1^.^4^ In addition, the selectivity of target aromatic monomers is highly dependent on the hydrogen transfer efficiency from the photocatalyst surface to aromatic monomeric radicals. For example, in the reaction system with a low hydrogen transfer efficiency, DB-one can be generated from the C–C coupling side reaction with the acetophenone radical, rather than the generation of acetophenone as the target aromatic monomer (Scheme 1).^3^ Therefore, it is important to improve the C_α_–H bond activation capability and enhance the hydrogen transfer efficiency on the photocatalyst surface to promote the reaction rate and selectivity to desirable products.

Based on the above discussions, the enhanced C_α_–H bond activation in lignin can facilitate the generation of C_α_ radical intermediates and improve the reaction rate of lignin conversion to aromatic monomers. The previous works demonstrated that the adsorbed hydroxyl radical (*OH) on the photocatalyst surfaces, as an important radical specie, can interact with substrate molecules to improve the C_α_–H bond activation through decreasing the BDE of C_α_–H bond.^19−24^ For example, in the photocatalytic oxidation of methane (CH_4_) process, the generation of *OH on the photocatalyst surfaces acts as the electrophilic radicals, which improve the oxidation capability of photocatalysts to promote the C_α_–H bond activation in CH_4_ to •CH_3_.^19−24^ Importantly, recent works demonstrated that the concentration of *OH on the photocatalyst surfaces can be controlled through methods such as adjusting the partial pressure of water in the reaction system, tuning the wavelength of light source, and optimizing the charge carriers separation efficiency, to improve the adsorption capability of substrate molecules on photocatalysts and promote the C_α_–H bond activation in CH_4_ oxidation,^21,25,26^ Inspired by the above research, optimization of the concentration of *OH on the photocatalyst surfaces could be able to improve the C_α_–H bond activation in lignin, thereby improving the reaction rate of lignin to desirable products.

The high hydrogen transfer efficiency from the photocatalyst surface to aromatic monomeric radicals can improve the selectivity of desirable products. Previous studies indicate that the hydrogen transfer efficiency on the photocatalyst surface is determined by the electron transfer efficiency.^27,28^ For example, Yu’s group worked on hydrogen evolution from water dissociation by using NiCuS_*x*_/TiO_2_ photocatalysts. They found that electron-rich active sites can facilitate the hydrogen transfer from interfacial S–H_ads_ bonds on NiCuS_*x*_ surfaces to improve the hydrogen evolution activity in the photocatalytic water splitting process.^27^ The thickness of nanophotocatalysts can affect electron migration. Previous works demonstrated that ultrathin nanosheet structures can facilitate electrons migration, leading to the formation of an electron pool, thereby improving the adsorbed hydrogen transfer efficiency on the photocatalyst’s surface.^29−33^ Yang’s group demonstrated that the reduction in the thickness of 2D ZnIn_2_S_4_ can shorten the diffusion distance of charge carriers from the interior to the surface and prolong their recombination time to improve the hydrogen evolution activity.^34^ Furthermore, Cao’s group demonstrated that 2D ZnIn_2_S_4_ photocatalysts can improve the surface area and provide more active sites compared with flower-like spheres and block ZnIn_2_S_4_ photocatalysts, therefore improving the hydrogen evolution activity.^35^ MoS_2_ as a promising cocatalyst can be loaded on the 2D ZnIn_2_S_4_ photocatalysts to further enhance the charge transfer efficiency and improve electron conductivity to boost the photocatalytic performance in hydrogen evolution.^36−41^ For example, MoS_2_ quantum dot loaded into the S vacancies in ZnIn_2_S_4_ monolayer can reduce the edge contact resistance and establish the intimate Zn–S bond interfaces, therefore facilitating the photogenerated electrons migration from ZnIn_2_S_4_ to MoS_2_ to improve the photocatalytic hydrogen reaction from water splitting.^36^ Zhang’s group prepared MoS_2-x_/ZnIn_2_S_4-x_ with dual S defects that can create an internal electric field between ZnIn_2_S_4-x_ and MoS_2-x_, therefore increasing the electron transfer rate and promoting efficient charge separation to enhance the hydrogen production.^42^

Inspired by the aforementioned works, this study mainly seeks to enhance the reaction rate of lignin conversion by optimizing the concentration of *OH on the photocatalyst surfaces to improve the C_α_–H bond activation in lignin and to increase the selectivity of desirable aromatic monomers by enhancing the electron transfer efficiency of photocatalysts to promote the hydrogen transfer efficiency. In detail, we optimized the amount of adsorbed *OH on the surfaces of photocatalysts through controlling the ratio of water in the reaction system to reduce the BDE of the C_α_–H bond and lengthen the C_α_–H bond in PP-ol, resulting in the enhancement of C_α_–H bond activation to facilitate the reaction rate of PP-ol to aromatic monomers. The elaborately designed half-unit-cell MoS_2_/ZnIn_2_S_4_ monolayer of around 1.7-nm-thick nanosheet can shorten the diffusion distance of charge carriers and facilitate the transfer of photogenerated electrons from the ZnIn_2_S_4_ monolayer to the MoS_2_ monolayer, therefore significantly improving the hydrogen transfer efficiency on the surface of photocatalysts to enhance the selectivity of desirable aromatic monomers from lignin conversion. As a result, PP-ol is completely converted to ∼85% of aromatic monomers after 1 h of visible light irradiation, which is the fastest reaction rate compared to previous works.

## 2 Experimental Section

### 2.1 Materials

All reagents were of analytical grade and used without further purification. Trisodium citrate dihydrate (99%+), thioacetamide (99%+), acetonitrile (CH_3_CN), Zn (NO_3_)_2_·6H_2_O (98%), InCl_3_ (anhydrous, 99.99%), and 1,2-dibenzoyl-1,4-butanedione (DB-one) were purchased from Fisher Scientific International, Inc. (NH_3_)_6_Mo_7_O_24_ (99%), phenol, acetophenone, sodium sulfide nonahydrate (98%+), sodium persulfate (99%), sodium sulfate (98.5%), D-mannitol, (3-bromopropyl) trimethoxysilane (BPTMOS), sodium hydrosulfide hydrate, TEMPO, and 5,5-dimethyl-1-pyrroline N-oxide (DMPO) were purchased from Sigma-Aldrich Co., Ltd. 2-Phenoxy-1-phenylethanol (PP-ol), coumarin (Cou), and 2-phenoxyacetophenone (PP-one) were purchased from Fluorochem Ltd. 2-(2-Methoxyphenoxy)-1-phenylethanol (MP-ol), 2-phenoxy-1-phenylpropane-1,3-diol (PPP-ol), 1-(3,4-dimethoxyphenyl)-2-(2-methoxyphenoxy) propane-1,3-diol (DMP-ol), and phenethoxybenzene (PEB) were purchased from BLD Pharmatech Ltd. Ethylene glycol (EG) was purchased from VWR International, LLC. Deionized (DI) water was produced by a CENTRA R200 Centralized Purification and Distribution Systems.

### 2.2 Preparation of X% MoS_2_/ZIS-300 Photocatalysts

**Figure 1:**
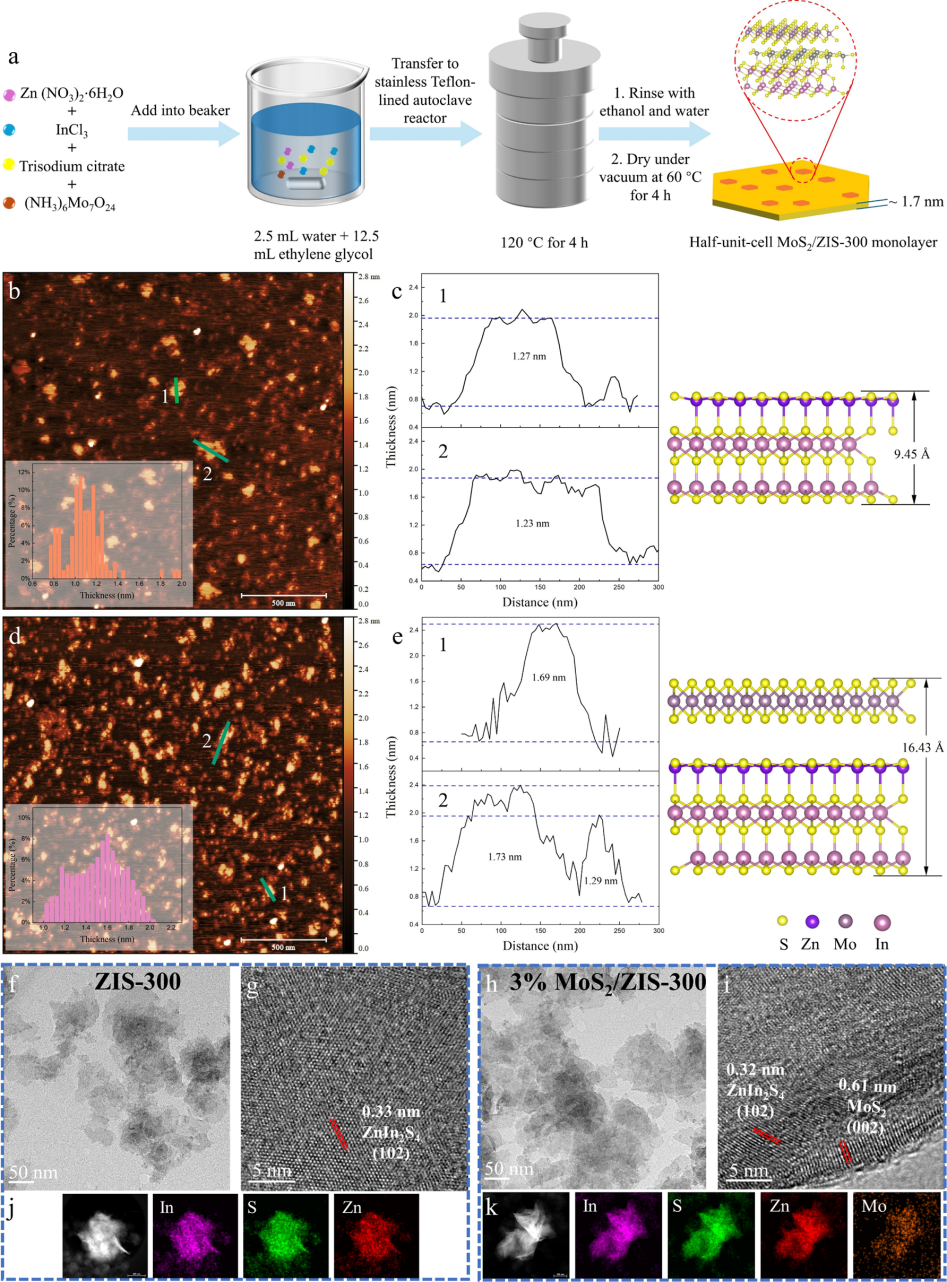
(a) Schematic diagram for the preparation of a half-unit-cell MoS_2_/ZnIn_2_S_4_ monolayer. AFM images and the corresponding height profile of ZIS-300 (b, c) and 3% MoS_2_/ZIS-300 (d, e). The inset graphs in (b) and (d) correspond to particle thickness distributions. TEM and HRTEM images, and element mappings of ZIS-300 (f, g, j) and 3% MoS_2_/ZIS-300 (h, i, k).

The half-unit-cell MoS_2_/ZIS-300 monolayer was prepared by a one-pot hydrothermal method (Figure 1a). Typically, 148.73 mg of Zn (NO_3_)_2_·6H_2_O, 221.18 mg of InCl_3_, 300 mg of trisodium citrate, and a certain amount of (NH_3_)_6_Mo_7_O_24_ were dissolved into the mixture of 2.5 mL of deionized water and 12.5 mL of ethylene glycol. After being ultrasonically dispersed for 10 min and vigorously stirred for 30 min at room temperature, 375 mg of thioacetamide was added to the solution. After being stirred for another 30 min, the mixture was transferred to a 25 mL stainless Teflon-lined autoclave reactor. The autoclave reactor was subsequently heated to 120 °C with a heating rate of 3 °C min^–1^ in an oven, and the temperature was maintained for 4 h. After natural cooling, the sample was collected by centrifugation (9000 rpm) and rinsed twice with ethanol and water, respectively. The solid samples were then dried under vacuum at 60 °C for 4 h. The obtained catalyst was labeled as x% MoS_2_/ZIS-300:0.5% MoS_2_/ZIS-300, 1.5% MoS_2_/ZIS-300, 3% MoS_2_/ZIS-300, 5% MoS_2_/ZIS-300, and 7.5% MoS_2_/ZIS-300, where x is the amount of trisodium citrate added in the solution. The half-unit-cell ZIS-300 monolayer was also synthesized via the same process but without (NH_3_)_6_Mo_7_O_24_. The pristine ZIS-0 was synthesized via the same process but without (NH_3_)_6_Mo_7_O_24_ and trisodium citrate. Labels “ZIS-300″ and “ZIS-0″ indicate the addition of trisodium citrate during the synthesis stage, with “ZIS-300″ meaning that 300 mg of trisodium citrate was added and “ZIS-0″ meaning that no trisodium citrate was added.

### 2.3 Photocatalytic Reaction Experiments

Photocatalytic reactions were performed in a customized quartz reactor with a cooling water jacket. A xenon arc lamp (manufactured by Perfect Light Company) equipped with a PE300BF-type light bulb and a 420 nm UV filter was applied as the light source. The position of the lamp was fixed during the entire experiment to maintain a constant light intensity of 0.35 W cm^–2^. Typically, 10 mg of reactants (PP-ol, MP-ol, PPP-ol, DMP-ol, or PP-one), and 10 mg of photocatalyst were dispersed in 5 mL of solvent (CH_3_CN and CH_3_CN/H_2_O mixture) in the reactor. The photocatalysts were dispersed by magnetic stirring, and the reactor was firmly sealed after 30 min of argon purge (10 mL min^–1^). The sealed reactor with 200 rpm of magnetic stirring was illuminated under visible light irradiation. The temperature of the reactor was kept around 20 °C with the cooling water system during the reaction. After the reaction, the solution was collected by centrifugation and methylparaben was added to the solution as the internal standard. The solution with internal standard was qualitatively analyzed by the gas chromatography–mass spectrometry (GC–MS, QP2010 SE, Shimadzu, Rxi-5 ms column) and quantitatively analyzed by gas chromatography (GC, GC-2010 plus, Shimadzu, Stabilwax-MS column). The program applied for GC analysis is injection temperature: 250 °C; column temperature program: 80 °C for 2 min, then increasing the temperature to 200 °C at a rate of 40 °C cm^–1^ and maintaining for 2 min, then heat up to 240 °C at a rate of 10 °C min^–1^, and holding the temperature for 9 min. The program applied for the GC–MS analysis is injection temperature: 280 °C; column temperature program: 80 °C for 2 min, then increasing the temperature to 270 °C at a rate of 10 °C min^–1^, then heat up to 300 °C at a rate of 30 °C min^–1^, and holding the temperature for 5 min. The calibration details are presented in Figure S1. The following equations were used to calculate the conversion rate of reactants, selectivity of products, and yields of products, respectively:
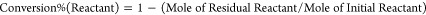
1
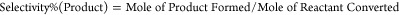
2
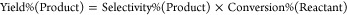
3

### 2.4 Characterization

Powder X-ray diffraction (XRD) patterns were recorded on a Bruker Phaser-D2 diffractometer with a Cu Kα X-ray source at a voltage of 40 kV and current of 40 mA. The morphologies of the as-prepared CdS were imaged by scanning electron microscopy (SEM, Zeiss Sigma VP) and high-resolution transmission electron microscopy (TEM/HRTEM, JEOL JEM-2100F). The BET results of the photocatalysts were recorded with a Quantachrome IQ sorption analyzer. The light absorption properties and bandgap results of prepared materials were measured using UV/vis diffuse reflectance spectroscopy (DRS) on a JASCO V-670 spectrophotometer equipped with an integration sphere in the spectral range of 200–1000 nm, and BaSO4 was used as the reflectance standard. The chemical states of each element in prepared samples and X-ray photoelectron valence band spectra (XPS-VB) were characterized by an X-ray photoelectron spectrometer (Thermo Fisher K-Alpha) with an Al Kα X-ray source. All peaks have been calibrated with the C 1s peak where the standard binding energy (B.E.) is 284.8 eV for the adventitious carbon source. The thickness of the samples was detected by atomic force microscopy (AFM, Bruker Multimode 8). The composition of each prepared catalyst was determined by EDS and inductively coupled plasma optical emission spectroscopy (ICP-OES, Varian Vista Pro). The emission intensity of CdS with or without PP-ol was measured by photoluminescence (PL, RF-6000, Shimadzu) under an excitation wavelength of 420 nm. The photoelectrochemical (PEC) measurements were performed in a standard three-electrode system by using an electrochemical workstation (CHI660E, Chenhua, shanghai) under visible light illumination in 20 mL of CH_3_CN and 30 mL of H_2_O with 30 mg of PP-ol and 0.2 M Na_2_ClO_4_.

## 3 Results and Discussion

### 3.1 Characterization of Half-Unit-Cell MoS_2_/ZIS-300 Monolayer

Half-unit-cell MoS_2_/ZIS-300 monolayer photocatalysts were prepared as shown in the schematic diagram in Figure 1a for the cleavage of the C_β_–O bond in lignin in this study. XRD, XPS, SEM, TEM, and AFM were conducted to confirm the successful synthesis of half-unit-cell MoS_2_/ZIS-300 monolayer photocatalysts. AFM was first employed to analyze the thicknesses of ZIS-300 and 3% MoS_2_/ZIS-300. These results demonstrate that both ZIS-300 and 3% MoS_2_/ZIS-300 are monolayer structures with half-unit-cell thickness. As shown in Figure 1b,c, the thickness of ZIS-300 is around 1.25 nm, aligning closely with the theoretical thickness of half-unit-cell ZnIn_2_S_4_ (0.95 nm). With the 2D MoS_2_ loading on the half-unit-cell ZIS-300 monolayer structure, the thickness increases to around 1.71 nm, matching the theoretical thickness of 1.64 nm (Figure 1d,e).

As shown in Figures 1f,h and S2, TEM and SEM images further reveal that ZIS-300 and 3% MoS_2_/ZIS-300 have ultrathin nanosheets. The HRTEM images of ZIS-300 (Figure 1g) show lattice fringes of 0.33 nm, corresponding to the (102) facet of ZnIn_2_S_4_. As shown in Figure 1i, the interplanar spacing of 0.32 and 0.61 nm in 3% MoS_2_/ZIS-300 represents the ZnIn_2_S_4_ (102) and MoS_2_ (002) facets, respectively, indicating the formation of a tight interface between ZIS-300 and MoS_2_.^40,41^ The element mappings (Figure 1j,k) show that Zn, In, S, and Mo were uniformly dispersed in the ultrathin structures. In summary, the half-unit-cell ZIS-300 and half-unit-cell MoS_2_/ZIS-300 monolayer structures were successfully synthesized.

The XRD patterns in Figure S3 show the main diffraction characteristic peaks of 21.59°, 27.69°, and 47.18°, corresponding to the crystallographic planes (006), (102), and (112) of hexagonal ZnIn_2_S_4_ crystal (JCPDS card no. 03–065–2023). However, the x% MoS_2_/ZIS-300 photocatalysts in XRD patterns cannot find the XRD peaks of MoS_2_, which is contributed to the low content of MoS_2_ within ZIS-300. Furthermore, the actual content of elements in x% MoS_2_/ZIS-300 was measured by ICP-OES. As shown in Table S1, the molar ratio of Mo to Zn shows an increasing trend, matching with the increasing amount of MoS_2_ in x% MoS_2_/ZIS-300.

XPS was carried out to investigate the chemical states of ZIS-300 and 3% MoS_2_/ZIS-300 photocatalysts. Figure S4a exhibits the full XPS spectrum of ZIS-300 and 3% MoS_2_/ZIS-300, including Zn, In, and S as the predominant elements. It is difficult to find the Mo peaks in the full XPS spectrum of 3% MoS_2_/ZIS-300, as the content of Mo in this photocatalyst is relatively low (Table S1). As shown in Figure S4b–d, Zn, In, and S elements in ZIS-300 and 3% MoS_2_/ZIS-300 were identified by high-resolution XPS spectra. The high-resolution XPS spectra of ZIS-300 show peaks of Zn 2p (1045.0 eV for Zn 2p_1/2_, and 1022.0 eV for Zn 2p_3/2_), In 3d (452.6 eV for In 3d_3/2_, and 445.0 eV for In 3d_5/2_), and S 2p (162.9 eV for S 2p_1/2_, and 161.6 eV for S 2p_3/2_), corresponding to Zn^2+^, In^3+^, and S^2–^, respectively. Compared to ZIS-300, all high-resolution XPS peaks of Zn 2p, In 3d, and S 2p in 3% MoS_2_/ZIS-300 are shifted to higher binding energy. These results are attributed to that MoS_2_ decorating on ZIS-300 monolayer facilitates the photogenerated electrons transmission from ZIS-300 to MoS_2_.^38^ As shown in Figure S4e, the high-resolution XPS spectrum of Mo 3d in 3% MoS_2_/ZIS-300 shows two weak peaks at 228.0 eV (Mo 3d_5/2_) and 230.3 eV (Mo 3d_3/2_), corresponding to Mo^4+^ as the main existence.^43^ These results demonstrate the 2D MoS_2_ decoration on the ZIS-300 monolayer that enhances the electron density. In summary, all characterizations prove that half-unit-cell MoS_2_/ZnIn_2_S_4_ monolayer photocatalysts were successfully prepared.

### 3.2 Improvement of Charge Carriers’ Transfer Efficiency and Visible Light Absorption Capability through Half-Unit-Cell MoS_2_/ZIS-300 Monolayer

**Figure 2:**
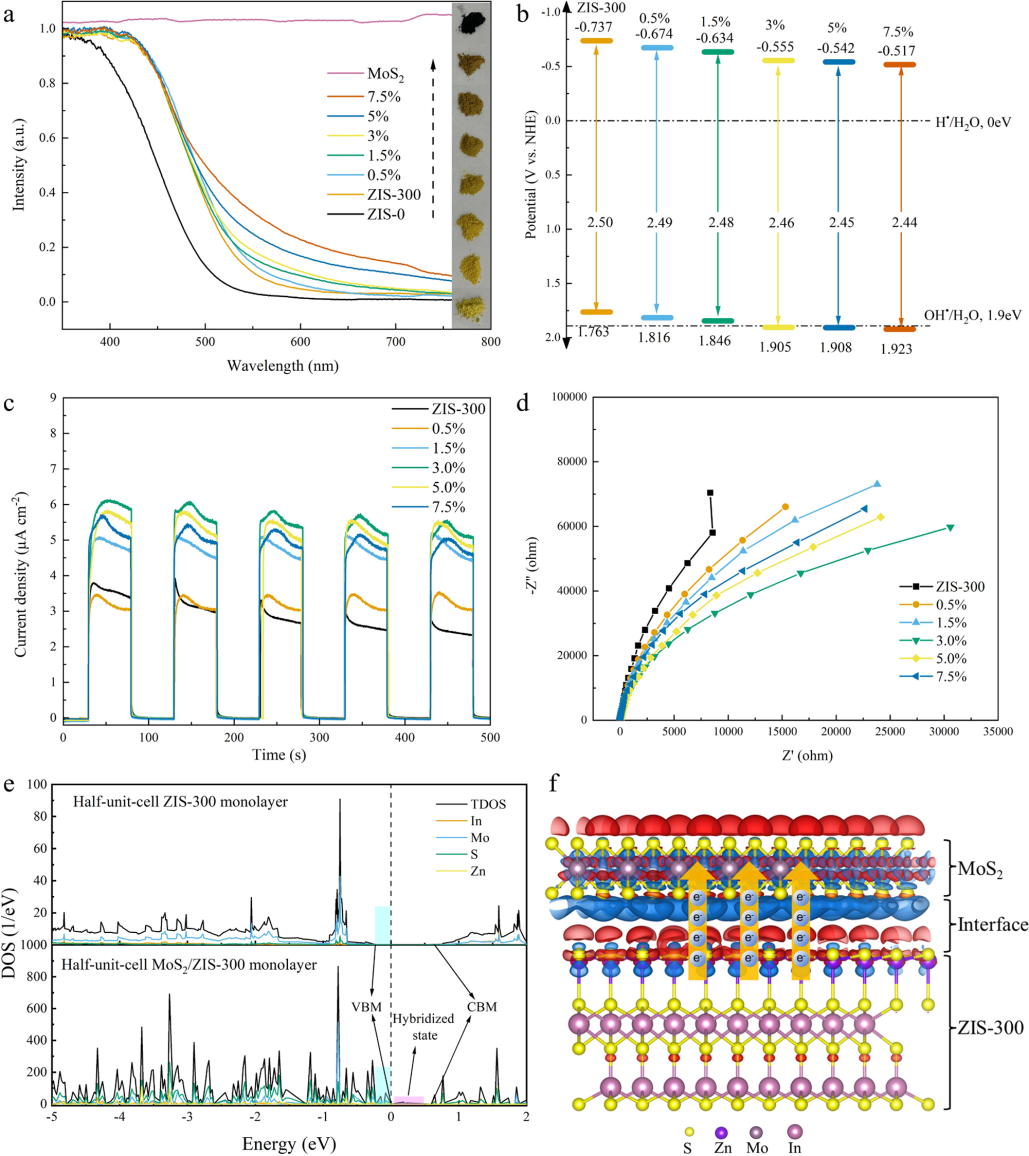
(a) UV–vis diffuse reflectance spectra of ZIS-0, ZIS-300, and x% MoS_2_/ZIS-300. (b) Band structure diagram of ZIS-300 and x% MoS_2_/ZIS-300. PEC properties of x% MoS_2_/ZIS-300: (c) transient photocurrent responses and (d) EIS spectra. (e) DOS of half-unit-cell ZIS-300 and half-unit-cell MoS_2_/ZIS-300. (f) Charge density distribution of half-unit-cell MoS_2_/ZIS-300. The blue and red parts indicate the accumulation and depletion of electrons, respectively.

Visible light absorption capability, photoredox capability, and photogenerated charge carrier transfer efficiency are important factors to the overall photocatalytic performance in lignin conversion. Herein, DRS, PL, and PEC measurements and density functional theory (DFT) calculations were employed to analyze these proprieties. DRS was conducted to investigate the visible light absorption properties and energy band positions of the photocatalysts. As shown in Figure 2a, the significant red shift by ZIS-0 to ZIS-300 demonstrates that half-unit-cell ZIS-300 monolayer structure can significantly improve the visible light absorption capability. The introduction of MoS_2_ in ZIS-300 can further improve solar-light-harvesting efficiency as demonstrated by the red shift with the ratio increase of MoS_2_ in MoS_2_/ZIS-300 from 0% to 7.5%.

To gain further insight into the bandgap edge and photoredox capability of photocatalysts, the bandgap (*E*_g_) and valence band potential (*E*_VB_) were calculated from DRS and XPS-VB results. As shown in Figures S5 and S6, the *E*_g_ slightly decreases from 2.50 eV in ZIS-300 to 2.44 eV in 7.5% MoS_2_/ZIS-300, and the E_VB_ gradually increases from 1.76 eV in ZIS-300 to 1.92 eV in 7.5% MoS_2_/ZIS-300. Based on the *E*_g_ and *E*_VB_ results, the conduction band potential (E_CB_) can be calculated using the equation (*E*_CB_ = *E*_VB_ – *E*_g_), as shown in Figure 2b. Typically, a higher *E*_CB_ represents a weaker reductive capability and a larger value of *E*_VB_ corresponds to a stronger oxidative capability.^1,3^ The x% MoS_2_/ZIS-300 monolayer shows an increase in *E*_VB_ and *E*_CB_, therefore showing stronger oxidative capability and weaker reductive capability with an increasing amount of MoS_2_ in MoS_2_/ZIS-300.

The density of states (DOS) is determined based on DFT calculation to further investigate the electronic properties and charge behaviors of the half-unit-cell ZIS-300 and half-unit-cell MoS_2_/ZIS-300 monolayer. As shown in Figure 2e, the DOS structure presents the valence band maximum (VBM) and conduction band minimum (CBM) for half-unit-cell ZIS-300 monolayer and half-unit-cell MoS_2_/ZIS-300 monolayer.^34,37,44−49^ The VBM increases from −0.21 eV in ZIS-300 to 0.02 eV in MoS_2_/ZIS-300, which is attributed to the d orbit of Mo and the p orbit of S (Figure S7). The CBM for both photocatalysts exhibits similar energy levels at 0.594 eV in MoS_2_/ZIS-300 and 0.595 eV in ZIS-300. These results demonstrate the enhanced electron donation-acceptance capability in the half-unit-cell MoS_2_/ZIS-300 monolayer.^47^ Furthermore, the pink area in Figure 2e shows a hybridized state beyond the Fermi levels in MoS_2_/ZIS-300. The partial density of states (PDOS) results in Figure S7 demonstrate that the p orbit of S and the s orbit of In trigger the hybridized state. This hybridization can act as an electron acceptor and thus effectively reduce the recombination time of photogenerated holes and electrons.^50^ In summary, these results indicate that the half-unit-cell MoS_2_/ZIS-300 monolayer can enhance photogenerated electron donation-acceptance capability and reduce the recombination time of photogenerated charge carriers.

The PEC and PL results of x% MoS_2_/ZIS-300, charge density difference distribution of half-unit-cell MoS_2_/ZIS-300, and work functions of MoS_2_ and ZIS-300 demonstrate that half-unit-cell MoS_2_/ZIS-300 monolayer can facilitate the photogenerated electrons migration from ZIS-300 to MoS_2_. The charge density difference distribution of half-unit-cell MoS_2_/ZIS-300 is simulated and is presented in Figure 2f. The blue part (the Mo and S atoms in MoS_2_) represents the charge carriers’ accumulation, and the red part (the Zn and S atoms in ZIS-300) corresponds to the charge carriers’ depletion. In the interface between ZIS-300 and MoS_2_, photogenerated electrons migrate from ZIS-300 to MoS_2_ following the arrow’s direction. The electron migration is driven by electron depletion in ZIS-300 of Zn and S atoms and electron accumulation capability in MoS_2_ of S and Mo atoms.^51^ As shown in Figure S8, the calculated work function of the MoS_2_ monolayer is 6.30 eV, while the calculated work function of ZIS-300 is 5.68 eV, which can further demonstrate that the photogenerated electrons transfer from ZnIn_2_S_4_ to MoS_2_. The PEC results demonstrated that 3% MoS_2_/ZIS-300 exhibits the highest photocurrent response capability (Figure 2c) and the smallest electrochemical impedance (Figure 2d) compared to other photocatalysts, refarming in the high charge carrier transfer efficiency and the low charge transfer resistance in 3% MoS_2_/ZIS-300. Furthermore, PL spectra of x% MoS_2_/ZIS-300 photocatalysts were measured under an excitation wavelength at 400 nm to investigate the recombination and migration dynamics of photogenerated charge carriers.^52^ As shown in Figure S9, all photocatalysts exhibit an emission peak at 547 nm. The emission intensity of 3% MoS_2_/ZIS-300 is weaker than that of the other photocatalysts, indicating a longer recombination time of photogenerated charge carriers in 3% MoS_2_/ZIS-300. In summary, all results demonstrate that 3% MoS_2_/ZIS-300 photocatalysts can improve the interaction between charge carriers and reactants and enhance the photocatalytic performance.

### 3.3 Photocatalytic Performance of Cleaving C_β_–O Bond in Lignin

**Scheme 2:**
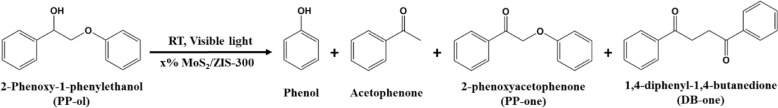
Photocatalytic Conversion of Lignin Model (PP-ol) to Phenol, Acetophenone, PP-One, and DB-One

PP-ol, as the dimeric lignin model compound with β–O–4 bond, was used as the reactant to evaluate the cleavage of C_β_–O bonds to aromatic monomers, because of its structural similarity to the lignin compounds with C_β_–O bonds.^2,3,13−15^ As shown in Scheme 2, PP-ol can be converted to generate acetophenone and phenol as the target aromatic monomers, whereas PP-one and DB-one are not desirable products. The mass spectrometry data from GC-MS and ^1^H and ^13^C NMR spectra were recorded to investigate the generated products in the photocatalytic conversion of PP-ol. As shown in Figure S10, the mass spectra of each generated product along with the corresponding standard mass spectra from the GC-MS library confirm that the generated products were acetophenone, phenol, PP-one, and DB-one. In addition, ^1^H and ^13^C NMR analyses for PP-ol, various products, and the reaction solution after visible light irradiation were performed to confirm that acetophenone and phenol are the major aromatic monomeric products in the photocatalytic fragmentation of PP-ol (detailed discussions are presented in Figure S11). The generation of PP-one is due to the dehydration of PP-ol, while the formation of DB-one is due to the coupling of two acetophenone radicals together through C–C coupled reaction.^2^ Previous works have demonstrated that PP-one, serving as the intermediate product, can be further converted into aromatic monomers.^3^ In order to exclude these possibilities, the PP-one and DB-one can be separately introduced as the reactants in the reaction system and are found not to be converted into aromatic monomers (entries 1–2 in Table 1), suggesting that they are ultimate byproducts rather than intermediate ones.

**Table 1:** Controlled Experiments for Fragmentation of the C_β_–O Bond^a^

				conversion/selectivity				
entry	catalyst	atmosphere	reactant	PP-ol	phenol	acetophenone	PP-one	DB-one
1	3% MoS_2_/ZIS-300	Ar	10 mg of PP-one	-	-	-	-	-
2	3% MoS_2_/ZIS-300	Ar	10 mg of DB-one	-	-	-	-	-
3	3% MoS_2_/ZIS-300	Ar	10 mg of PP-ol	100%	86.6%	82.3%	11.8%	3.1%
4	3% MoS_2_/ZIS-300	N_2_	10 mg of PP-ol	100%	85.4%	84%	13.1%	4.2%
5	3% MoS_2_/ZIS-300	He	10 mg of PP-ol	100%	84.1%	79.4%	14.9%	2.9%
6	3% MoS_2_/ZIS-300	Air	10 mg of PP-ol	100%	-	-	100%	-
7	3% MoS_2_/ZIS-300	O_2_	10 mg of PP-ol	100%	-	-	100%	-
8	ZIS-0	Ar	10 mg of PP-ol	30.9%	12.5%	11.1%	17.9%	-
9	ZIS-300	Ar	10 mg of PP-ol	88.5%	70.5%	53.6%	18.7%	10%
10	MoS_2_	Ar	10 mg of PP-ol	-	-	-	-	-

a Typical reaction condition: lignin model compound PP-ol is 10 mg, photocatalyst is 10 mg, solvent (CH_3_CN/H_2_O (v/v = 2/3)) is 5 mL, Ar is at 1 atm, visible light power is 0.35 W cm^–2^, 1 h.

The atmosphere in the reaction system is an important factor in determining the selectivity of aromatic monomers for lignin conversion. As shown in entries 3–5 in Table 1, the PP-ol is completely converted and the selectivities of acetophenone and phenol are around 84% after 1 h of visible light irradiation in different inert atmospheres (N_2_, He, and Ar). However, in the presence of O_2_, PP-ol is completely converted to PP-one (entries 6–7 in Table 1), as O_2_ can form reactive oxygen species (e.g., •O^2–^) to drive the oxidation of PP-ol to PP-one.^53^ Herein, the following experiments were performed under an Ar inert atmosphere.

#### 3.3.1 Improvement of the Reaction Rate through Enhanced Hydrogen Transfer Efficiency via Half-Unit-Cell MoS_2_/ZIS-300 Monolayer

The half-unit-cell ZIS-300 monolayer photocatalysts can supply more active sites and enhance the visible light absorption capability to improve the conversion rate of PP-ol to some degree. As shown in entry 8 in Table 1, ZIS-0 with marigold structure (Figure S2) can convert 30.9% of PP-ol to 12.5% of phenol and 11.1% of acetophenone as desirable products, and 17.9% of PP-one is produced as a byproduct after 1 h of visible light irradiation. In contrast, when the thickness of the developed half-unit-cell ZIS-300 monolayer is reduced to ∼1.2 nm, the conversion rate of PP-ol is significantly improved from 30.9% to 88.5%, and the yields of the desirable products are improved from 12.5% to 70.5% for phenol and from 11.1% to 53.6% for acetophenone after 1 h of visible light irradiation (entry 9 in Table 1). This improvement is attributed to the enhanced visible light absorption capability of the half-unit-cell ZIS-300 monolayer with a thickness of ∼1.2 nm to improve the photogenerated charge carriers’ migration efficiency, as demonstrated by the DRS results in Figure 2a, and to increase the surface area (120.2 m^2^/g) exposing more active sites, as showed by the BET results in Figure S12. Although the half-unit-cell ZIS-300 monolayer can increase the PP-ol conversion, 10% DB-one is still produced as the C–C coupled byproduct. The 10% of DB-one byproduct significantly reduces the selectivity of acetophenone as the desirable products. The generation of DB-one is due to the poor hydrogen transfer efficiency on the surfaces of half-unit-cell ZIS-300, where the acetophenone radical cannot effectively obtain hydrogen on the surface of photocatalysts. As a result, two acetophenone radicals trigger the C–C coupled reaction to generate DB-one.

**Figure 3:**
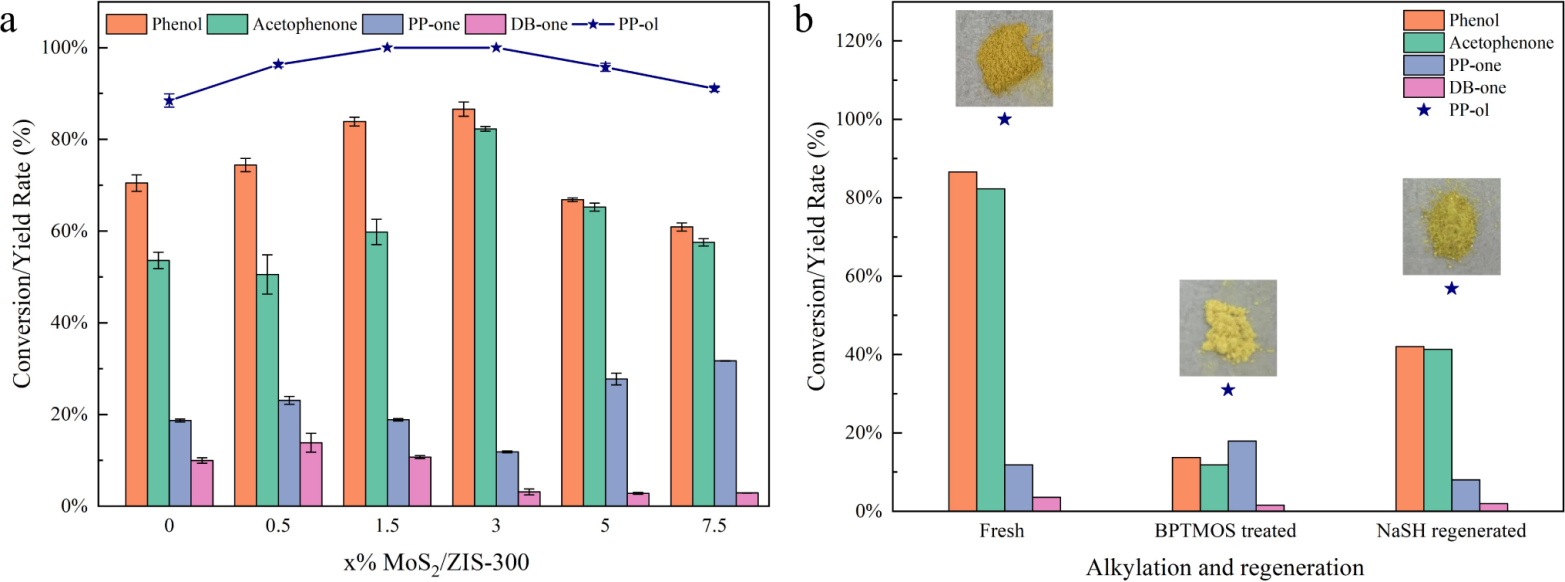
Conversion rate of PP-ol and the yields of products with (a) x% MoS_2_/ZIS-300 and (b) BPTMOS-treated and NaSH-regenerated 3% MoS_2_/ZIS-300. Reaction condition: lignin model compound PP-ol is 10 mg, photocatalyst is 10 mg, solvent (CH_3_CN/H_2_O (v/v = 2/3)) is 5 mL, Ar is at 1 atm, and visible light power is 0.35 W cm^–2^, 1 h.

The designed 2D MoS_2_-loaded half-unit-cell ZIS-300 monolayer can improve the hydrogen transfer efficiency to reduce the yields of DB-one, and can provide appropriate photoredox capability to improve the cleavage of C_β_–O bond in PP-ol and decrease the selectivity of PP-one as the overoxidized byproduct. As shown in Figure 3a, with the increasing amount of MoS_2_ in x% MoS_2_/ZIS-300 from 0% to 3%, the conversion rate of PP-ol is gradually increased from 88.5% to 100% and the yields of aromantic monomers increased from 70.5% to 86.6% for phenol and from 53.6% to 82.3% for acetophenone after 1 h of visible light irradiation. The yields of DB-one significantly decrease from 10% to 2.9%, and the yields of PP-one drop from 18.7% to 11.8% from ZIS-300 to 3% MoS_2_/ZIS-300. In comparison with previous works in the literature,^1−3,14,17^ our developed 3% MoS_2_/ZIS-300 shows the fastest conversion rate of PP-ol to aromatic monomers (Table S2). When the ratio of MoS_2_ to ZIS-300 is higher than 3%, the conversion rate and the selectivity to acetophenone and phenol gradually decrease, with a notable increase in the PP-one byproduct. These declines might be attributed to the increased oxidation capability from excess amount of MoS_2_ causing the overoxidation of PP-ol to PP-one, as the E_VB_ increases from 1.905 eV in 3% MoS_2_/ZIS-300 to 1.923 eV in 7.5% MoS_2_/ZIS-300. The result in entry 10 in Table 1 shows that the individual MoS_2_ cannot convert PP-ol, suggesting that MoS_2_ is a cocatalyst to improve the charge carrier transfer efficiency and hydrogen transfer efficiency rather than acting as a photocatalyst. The 3% MoS_2_/ZIS-300 photocatalyst shows the best photocatalytic performance than other photocatalysts, as it shows high hydrogen transfer efficiency to reduce the generation of DB-one as the C–C coupled byproduct, and provide an appropriate photoredox capability (band structure results in Figure 2b) to enhance the reaction rate of PP-ol and decrease the overoxidation of PP-ol to PP-one byproduct. In addition, it is worth mentioning that the 3% MoS_2_/ZIS-300 ultrathin nanosheet can keep uniform dispersibility in the reaction system after 48 h, as shown in Figure S13. The uniform dispersion increases the contact between photocatalysts and PP-ol, thereby improving the rate of PP-ol conversion.

The sulfur moieties on the half-unit-cell MoS_2_/ZIS-300 monolayer could potentially act as the active sites to promote C_α_–H bond activation in PP-ol, therefore enhancing the conversion rate of PP-ol to aromatic monomers. To confirm the role of sulfur moieties for activation of C_α_–H bonds in PP-ol conversion to aromatic monomers, BPTMOS was used to inhibit the sulfur moieties on the surfaces of photocatalysts through alkylating 3% MoS_2_/ZIS-300 in cyclohexane solution. As shown in Figure 3b, after inhibition of sulfur moieties through BPTMOS treatment, 3% MoS_2_/ZIS-300 shows significantly reduced photocatalytic activity. The conversion rate of PP-ol decreased from 100% to 37% and the selectivity of desirable products decreased from around 85% to around 44%. To further confirm the role of sulfur moieties for activation of the C_α_–H bond in PP-ol conversion, the photocatalytic activity of MoS_2_/ZIS-300 was regenerated by using NaSH to remove the BPTMOS from the treated 3% MoS_2_/ZIS-300 in the aqueous solution. After regeneration, the conversion rate of PP-ol is restored to 60% and the selectivity of aromatic monomers is increased to around 74% after 1 h of visible light irradiation. These results confirm that the sulfur moieties as the active sites on the surface of MoS_2_/ZIS-300 photocatalysts can activate the C_α_–H bond in PP-ol and cleave the C_β_-O bond to the aromatic monomers.

#### 3.3.2 Improvement of the C_α_–H Bond Activation in PP-Ol via the Adsorbed Hydroxyl Radical on Photocatalyst Surfaces

In the process of C_β_–O bond cleavage to aromatic monomers, the formation of C_α_ radical intermediate is a critical step, as C_α_ radical intermediate can significantly reduce the BDE of C_β_–O bond from 55 kcal mol^–1^ in PP-ol to 7.8 kcal mol^–1^,^4^ promoting the production of phenol and acetophenone. The formation of the C_α_ radical intermediate can be determined by the C_α_–H bond activation in PP-ol. Our experimental results and DFT simulation indicate that the hydroxyl radical (•OH) could be generated from the photocatalytic water oxidation process and adsorbed on the MoS_2_/ZIS-300 surface to form *OH, thereby significantly improving the C_α_–H bond activation in PP-ol and increasing the reaction rate of PP-ol conversion.

**Figure 4:**
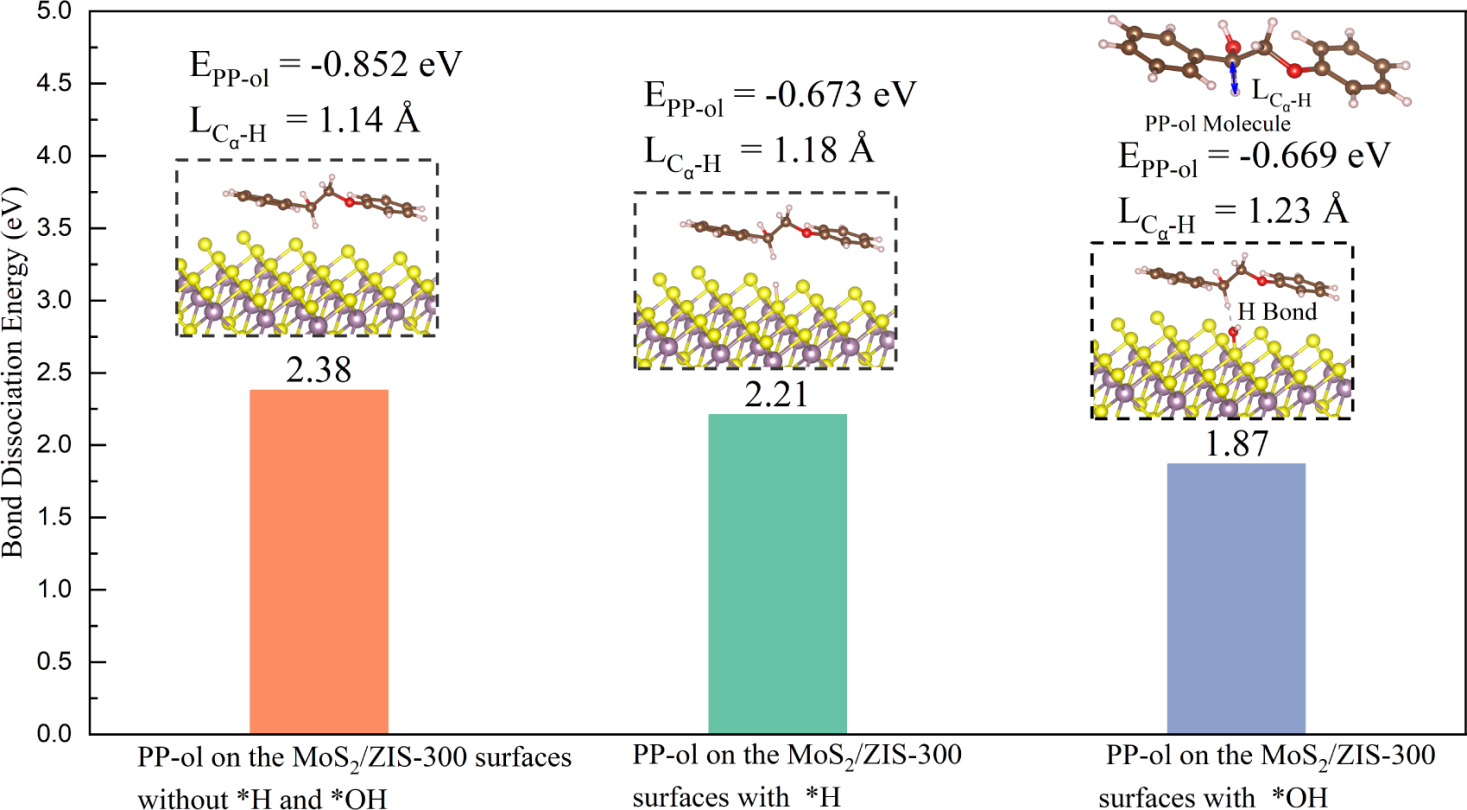
DFT calculation for E_PP-ol_, BDE of C_α_–H bond, and L_Cα–H_ on three conditions: (1) MoS_2_/ZIS-300 surfaces without *H and *OH, (2) MoS_2_/ZIS-300 surfaces with *H, and (3) MoS_2_/ZIS-300 surfaces with *OH.

As shown in the literature, *OH and adsorbed proton (*H) on photocatalysts surfaces can improve the C_α_–H bond activation in the oxidation of methane to methanol, C–C coupling of methanol into ethylene glycol, etc.^22,26,54,55^ In our reaction system, *OH and *H might have the potential to promote C_α_–H bond activation in PP-ol molecules and facilitate the generation of aromatic monomers. To assess this possibility, DFT simulation was first conducted to investigate the adsorption energy of PP-ol (E_PP-ol_), C_α_–H bond lengths (L_Cα–H_), and the BDE of C_α_–H bonds in PP-ol under three conditions, which are (1) MoS_2_/ZIS-300 surfaces without *H and *OH, (2) MoS_2_/ZIS-300 surfaces with *H, and (3) MoS_2_/ZIS-300 surfaces with *OH. As shown in Figures 4 and S14, the calculated E_PP-ol_ for conditions (2) and (3) are −0.673 eV and −0.669 eV, respectively, higher than the E_PP-ol_ for condition (1) at −0.852 eV. These mean that the presence of *OH or *H can weaken the interaction between PP-ol and MoS_2_/ZIS-300 surface, promoting the desorption of C_α_ radical intermediate after the C_α_–H bond activation, therefore providing vacant active sites for the activation of subsequent PP-ol molecule.^56,57^ The L_Cα–H_ and BDE of the C_α_–H bond were further calculated through DFT calculation. As shown in Figure 4, the L_Cα–H_ for condition (3) is 1.23 Å, longer than 1.18 Å for condition (2) and 1.14 Å for condition (1). The BDE of the C_α_–H bond in PP-ol is 2.38 eV for condition (1) and 2.21 eV for condition (2), and the BDE of the bond significantly decreases to 1.87 eV for condition (3). Furthermore, •OH scavengers were conducted to investigate the impact of the photocatalytic performance of PP-ol conversion to aromatic monomers. As shown in Figure 6a, 0.1 mM Cou, a •OH scavenger, was added to the reaction system, resulting in a decrease in the conversion rate of PP-ol from 100% to 65.3% after 1 h of visible light irradiation and a significant increase in the selectivity of PP-one from 11.8% to 53.0%. Both DFT simulations and •OH scavenger results demonstrate that *OH on the surfaces of MoS_2_/ZIS-300 from water oxidation can facilitate the C_α_–H bond activation to form C_α_ radical intermediates, promoting PP-ol conversion to aromatic monomers.

**Figure 5:**
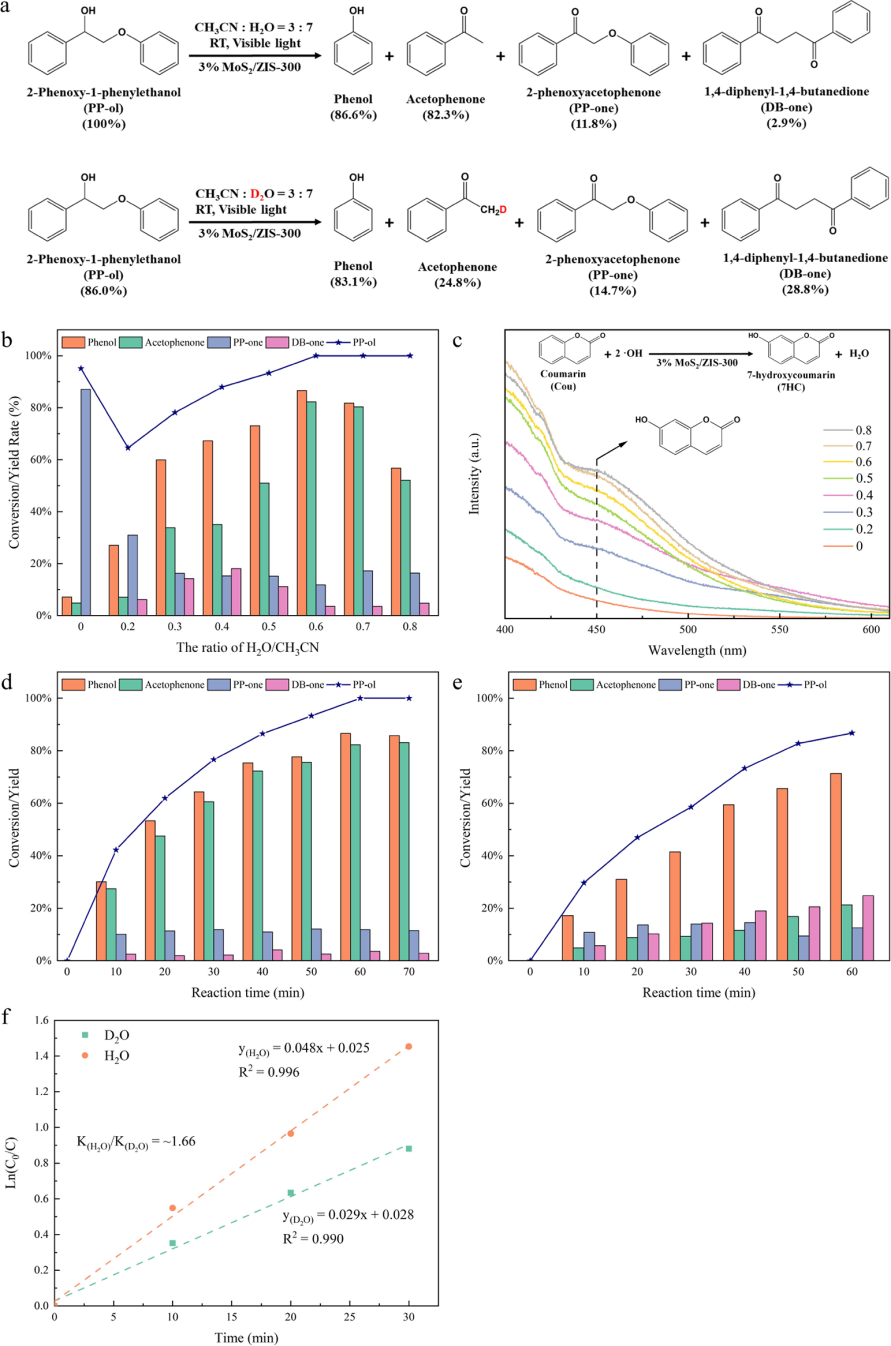
(a) Photocatalytic conversion of PP-ol in H_2_O–CH_3_CN and D_2_O–CH_3_CN solvent conditions via 1 h of visible light irradiation. (b) Effect of water amount on the conversion rate of PP-ol and the yields of products. (c) Fluorescence spectra of coumarin solution with different ratios of H_2_O/CH_3_CN. Conversion rate of PP-ol and the yields of products with (d) effect of reaction time on conversion rate under 3% MoS_2_/ZIS-300 in H_2_O condition and (e) effect of reaction time on conversion rate under 3% MoS_2_/ZIS-300 in D_2_O condition. (f) The calculated KIE under H_2_O and D_2_O conditions. Reaction condition: lignin model compound PP-ol is 10 mg, photocatalyst is 10 mg, solvent (CH_3_CN/H_2_O (v/v = 2/3)) is 5 mL, Ar is at 1 atm, visible light power is 0.35 W cm^–2^, 1 h.

The concentration of *OH on the MoS_2_/ZIS-300 surfaces can be controlled by optimizing the ratio of H_2_O/CH_3_CN to H_2_O on the reaction system. As shown in Figure 5b, in the water-free system, the conversion rate of PP-ol is relatively high at 95%, but around 85% of the reaction product is PP-one as the byproduct after 1 h of visible light irradiation. The low selectivity of aromatic monomers is due to the absence of *OH generation to activate the C_α_–H bonds and cleave the C_β_–O bonds in PP-ol. When the ratio of H_2_O/CH_3_CN in the system increased from 0 to 0.6, the selectivity to desirable aromatic monomers gradually increases from around 15% to around 85%, as the addition of water to the reaction system significantly increases the concentration of *OH. The interesting phenomenon is that the conversion rate of PP-ol is reduced from 95% in the water-free system to 64.5% when the water ratio is low (0.2 of H_2_O/CH_3_CN). However, when the water ratio is greater than 0.6, the conversion rate of PP-ol is increased to 100%. Under the water-free system, the photogenerated charges entirely interact with PP-ol, thereby increasing the conversion rate of PP-ol. However, under the low ratio of the H_2_O/CH_3_CN system, the low conversion rate of PP-ol is because water consumes a portion of the photogenerated charges, and the generated *OH are insufficient to effectively improve the activation of C_α_–H bonds in PP-ol. The ratio of H_2_O to CH_3_CN at 0.6 achieves the best photocatalytic performance after 1 h of visible light irradiation, where the conversion rate of PP-ol is 100% and the selectivities of acetophenone and phenol are 82.3% and 86.6%, respectively. However, when the ratio of H_2_O/CH_3_CN reaches 0.8, despite the complete conversion of PP-ol, products in the system fail to achieve stoichiometric balance due to the limited solubility of the reactant and generated products.^14^ The improvement of photocatalytic performance is attributed to the increased concentration of *OH on the surfaces of MoS_2_/ZIS-300,^21,25,26^ To evaluate it, PL with Cou as a molecular probe was conducted.^58^ In detail, the generated *OH from water oxidation can be reacted with Cou to generate 7HC (the inset equation in Figure 5c), and the generated 7HC can be identified by PL. As shown in Figure 5c, the fluorescence intensity of 7HC is increased with the ratio of water increasing from 0 to 0.8, indicating that the amount of *OH is increased with the ratio of water in the reaction system. All results demonstrate that the increasing amount of *OH through the ratio of water in the reaction system can significantly improve the reaction rate to achieve complete conversion of PP-ol and enhance the selectivity of target aromatic monomers.

The H_2_O/D_2_O kinetics experiment and deuterium kinetic isotope effect (KIE) results confirm that the generation of adsorbed *OH on the surfaces of MoS_2_/ZIS-300 is an important step in the activation of C_α_–H bonds in PP-ol and the cleavage of the C_β_–O bond process. Figure 5d,e shows the time-on-course conversion of PP-ol in the H_2_O and D_2_O systems, respectively. In the D_2_O reaction system, the conversion rate of PP-ol is only 86%, while the PP-ol conversion is 100% in the H_2_O system after 1 h of visible light irradiation. This difference is attributed to the higher BDE of the O–D bond in D_2_O compared to the BDE of the O–H bond in H_2_O,^59,60^ leading to a decrease the concentration of *OD on the MoS_2_/ZIS-300 surface, and then reducing the C_α_–H bond activation in PP-ol by *OD. Based on the H_2_O/D_2_O isotopic experiments, the KIE can be calculated to confirm the importance of O–H bond dissociation to generate *OH on the conversion rate of PP-ol. Generally, a larger KIE value indicates that the process plays a crucial role in the overall reaction performance.^59,60^ As shown in Figure 5f, the calculated KIE value (=/) of around 1.66 confirms that the generation efficiency of *OH on the surfaces of MoS_2_/ZIS-300 is an important step in the C_α_–H bond activation for the conversion rate of PP-ol to aromatic monomers.

In the photocatalytic reaction system, water provides not only the *OH on the MoS_2_/ZIS-300 surfaces to improve the C_α_–H bonds activation in PP-ol but also sufficient external hydrogen source to facilitate the hydrogen transfer efficiency,^18^ to enhance the formation of acetophenone and prevent the generation of DB-one as the C–C coupled byproduct. Both DB-one and acetophenone are competitively generated from acetophenone radicals. As discussed in Section 3.3.1, the selectivity of DB-one and acetophenone is determined by the hydrogen transfer efficiency of the reaction system. If a reaction system has a high hydrogen transfer efficiency, the acetophenone radical can easily obtain hydrogen and convert to acetophenone; otherwise, it generates DB-one through C–C coupled side reaction.^3^ We found that a high ratio of water in the reaction system can provide sufficient external hydrogen donors to facilitate hydrogen transfer efficiency and prevent the generation of DB-one. As shown in Figure 5b, with increasing the water ratio from 0.2 to 0.6, the selectivity of DB-one significantly decreases from 20% to 2.9%, and the selectivity of acetophenone notably increased from 10.9% to 82.3%. These results demonstrate that the increased water ratio can improve the hydrogen transfer efficiency in the reaction system, thereby significantly enhancing the selectivity of acetophenone and hindering the generation of DB-one.

The isotopic experiments of H_2_O/D_2_O were further used to investigate the impact of the hydrogen transfer efficiency on the selectivity of DB-one and acetophenone. Previous research has indicated that the hydrogen transfer efficiency in the D_2_O system is lower than that in the H_2_O system.^61^ As shown in Figure 5a, the selectivity of acetophenone in the D_2_O system significantly decreases from 82.3% in the H_2_O system to 24.8%, while the selectivity of DB-one dramatically increases from 2.9% to 28.8% after 1 h of visible light irradiation. In addition, the GC-MS results in the isotopic experiment of H_2_O/D_2_O further identify that the hydrogen in the formation of acetophenone is obtained from water, while the hydrogen in the formation of phenol is sourced from PP-ol itself. As shown in Figure S15 and S16, the MS peak of acetophenone shifts from 120 *m*/*z* in the H_2_O system to 121 *m*/*z* in the D_2_O system, indicating that one hydrogen atom in acetophenone is deuterium. Furthermore, the MS peak of acetophenone at 43 *m*/*z* in the H_2_O system shifts to 44 *m*/*z* in the D_2_O system, corresponding to the location of deuterium in its methyl group O=C–CH_3_(CH_2_D). These results demonstrate that the hydrogen obtained in the formation of acetophenone is from water. However, the MS peaks of phenol in the D_2_O system and H_2_O system remain identical, indicating that the hydrogen in the generation of phenol is from PP-ol itself by self-hydrogen transfer process.^3^

### 3.4 Mechanism of C_β_–O Bond Cleavage

**Figure 6:**
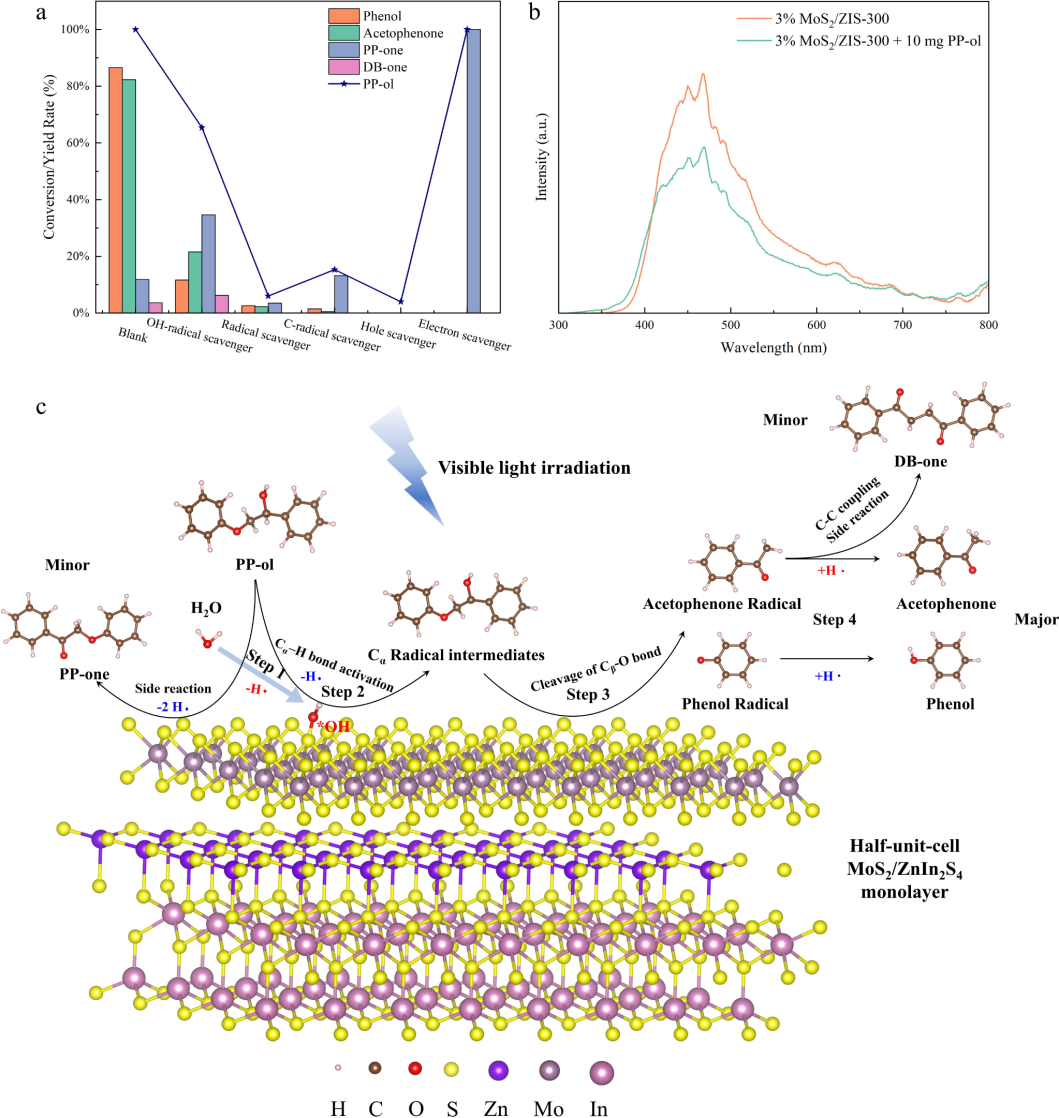
(a) Controlled experiments by 3% MoS_2_/ZIS-300 with different scavengers. Reaction conditions: lignin model compound PP-ol is 10 mg, 3% MoS_2_/ZIS-300 is 10 mg, solvent (CH_3_CN/H_2_O (v/v = 2/3)) is 5 mL, Ar is at 1 atm, visible light power is 0.35 W cm^–2^, 1 h. Hole scavengers: 20 mg of Na_2_S and 10 mg of Na_2_SO_3_; electron scavengers: 30 mg of Na_2_S_2_O_8_; radical scavengers: 30 mg of DMPO; C-centered radical scavengers: 30 mg of TEMPO; hydroxyl radical scavengers: 0.1 mM Cou. (b) PL emission spectra of 3% MoS_2_/ZIS-300 with and without PP-ol under visible light irradiation (excitation wavelength at 420 nm). (c) Proposed mechanism of C_β_–O bond fragmentation in the photocatalytic conversion of PP-ol over a 3% MoS_2_/ZIS-300 photocatalyst.

In the PP-ol conversion process, the photogenerated holes (h^+^) and electrons (e^–^) and the formation of C_α_ radical intermediates through activation of C_α_–H bond in PP-ol play critical roles in improving the conversion rate to desirable aromatic monomers. The cleavage of C_β_–O bond in PP-ol follows the radical intermediate mechanism.^4^ Hence, 30 mg of DMPO radical scavenger was added to the photocatalytic system to hinder the generation of radical intermediates and evaluate their impact on the photocatalytic performance. As shown in Figure 6a, the conversion rate of PP-ol is significantly suppressed, indicating that the formation of radical intermediates is an important factor to cleave the C_β_–O bond and generate acetophenone and phenol. Previous works have shown that C_α_ radical intermediates from activation of C_α_–H bond in PP-ol are the major radical intermediates to determine the conversion rate and selectivity of aromatic monomers.^4,13,15,18^ Hence, 30 mg of TEMPO was added to hinder the generation of C_α_ radical intermediates, as TEMPO preferentially captures C-centered radicals in protein modification^62^ and production of unsymmetrical disulfides.^63^ As shown in Figure 6a, only 15.3% of PP-ol converts to 13.2% of PP-one byproduct after 1 h of visible light irradiation. The results indicate that the formation of the C_α_ radical intermediate through activation of the C_α_–H bond in PP-ol is essential in the cleavage of the C_β_–O bond to acetophenone and phenol.

The PL spectra in Figure 6b confirm that the interaction between photogenerated charge carriers and PP-ol is an important step in the cleavage of C_β_–O bond to aromatic monomers. The emission intensity of the excited state of 3% MoS_2_/ZIS-300 is relatively high without PP-ol, but it significantly decreases in the presence of PP-ol. These results demonstrate that the photogenerated charge carriers, including both h^+^ and e^–^, can interact with PP-ol, which prolongs the recombination time of charge carriers and improves the efficiency of photogenerated charge carriers’ utilization. Furthermore, hole scavenger and electron scavenger were employed to separately hinder h^+^ and e^–^ in the reaction system, aiming to assess their respective impacts on the overall reaction. The addition of the hole scavengers (20 mg of Na_2_S and 10 mg of Na_2_SO_3_) can significantly decrease the photocatalytic conversion rate of PP-ol at only 4% (Figure 6a), as the suppressed h^+^ cannot oxidize water to generate *OH, therefore hindering the activation of C_α_–H bonds in PP-ol. Electron scavengers (30 mg of Na_2_S_2_O_8_) can promote the complete conversion of PP-ol to PP-one and entirely hinder the generation of acetophenone and phenol. In this process, the generated *OH by h^+^ can still activate the C_α_–H bonds to form C_α_ radical intermediates, but it is further oxidized to PP-one rather than cleavage of C_β_–O bonds. The results demonstrate that the cleavage of the C_β_–O bonds is entirely dependent on e^–^. In summary, the formation of C_α_ radical intermediate via activation of C_α_–H bonds, h^+^, and e^–^ collectively promotes the efficient conversion of PP-ol into desirable aromatic monomers.

Based on the above results, Figure 6c proposes a plausible mechanism for the cleavage of C_β_–O bonds in PP-ol. Under visible light irradiation, water can be first dissociated to generate •OH and adsorb on the surfaces of MoS_2_/ZIS-300 to form *OH (Step 1). In step 2, the C_α_–H bonds in PP-ol are effectively activated onto the surfaces of MoS_2_/ZIS-300 with *OH, generating C_α_ radical intermediates. In this process, the improved activation of C_α_–H bonds in PP-ol on the surfaces of MoS_2_/ZIS-300 with *OH is attributed to a reduction in the BDE of C_α_–H bonds from 2.38 eV on the surfaces of MoS_2_/ZIS-300 to 1.87 eV and an increase in the L_Cα–H_ from 1.14 Å on the surfaces of MoS_2_/ZIS-300 to 1.23 Å (Figure 4). However, in this step, a side reaction is observed where PP-ol is oxidized to the PP-one byproduct on the surfaces of MoS_2_/ZIS-300 without *OH. This side reaction can be explained by our experimental result, where PP-ol is converted to much higher yields of PP-one in the water-free system (Figure 5b). In step 3, the formed C_α_ radical intermediates effectively cleave the C_β_–O bond by e^–^, resulting in the generation of acetophenone and phenol radicals. The effective cleavage of the C_β_–O bonds in C_α_ radical intermediate is attributed to the reduction of the BDE of C_β_–O bonds from 55 kcal mol^–1^ in PP-ol to 7.8 kcal mol^–1^.^4^ In step 4, both the acetophenone and phenol radicals obtain hydrogen on the surfaces of MoS_2_/ZIS-300. The isotopic experiments demonstrate that acetophenone radicals obtain hydrogen from water dissociation, while the phenol radicals acquire hydrogen from C_α_–H bond activation in PP-ol. However, in the reaction system with low hydrogen transfer efficiency, DB-one is generated as a byproduct through the self-coupling of acetophenone radicals in Step 4.

### 3.5 Photostability and Feasibility of Half-Unit-Cell MoS_2_/ZIS-300 Monolayer in the Lignin Conversion System

**Scheme 3:**
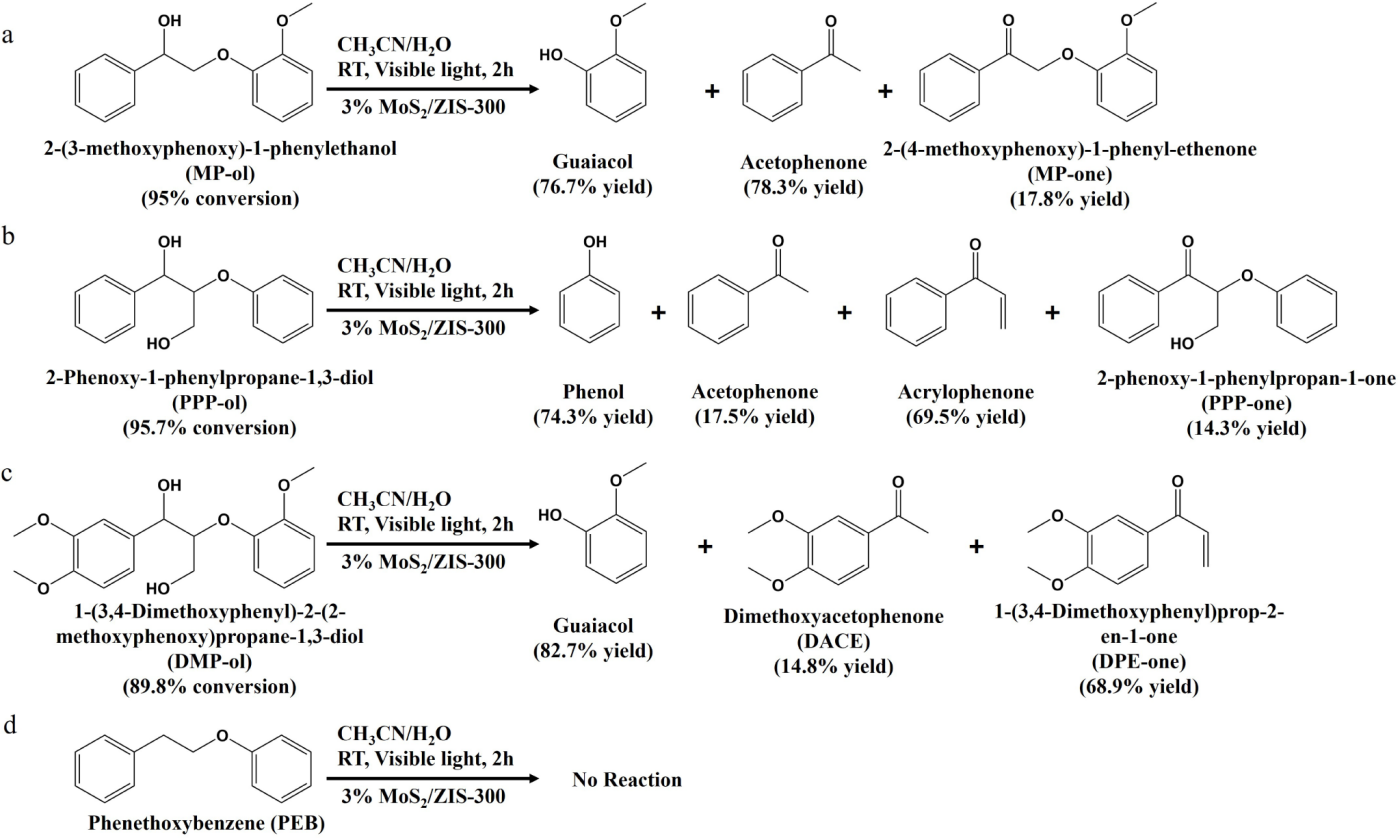
Conversion of Different Lignin Models to Desirable Aromatic Monomers after 2 h of Visible Light Irradiation (a) MP-ol;(b) PPP-ol;(c) DMP-ol;(d) PEB. Reaction conditions: lignin model compounds are 10 mg, 3% MoS_2_/ZIS-300 is 10 mg, solvent (CH_3_CN/H_2_O (v/v = 2/3)) is 5 mL, Ar is at 1 atm, and visible light power is 0.35 W cm^–2^, 2 h.

The feasibility and photostability of MoS_2_/ZIS-300 photocatalysts are important factors influencing their long-term application in the fragmentation of lignin into aromatic monomers. In this section, these properties can be investigated by depolymerization of different typical β-O-4 lignin models and real lignin, and the recycled experiments. The feasibility of 3% MoS_2_/ZIS-300 in the depolymerization of different typical β-O-4 lignin models was first evaluated. The methoxy group represents a significant moiety in lignin, and the cleavage of the C_β_–O bond in the lignin model containing a methoxy group is essential to evaluate the feasibility of the MoS_2_/ZIS-300 monolayer photocatalysts for lignin conversion. Therefore, MP-ol as the methoxy-substituted lignin model compound was used to examine the potential of developed MoS_2_/ZIS-300 for lignin conversion. As shown in Scheme 3 and Figure S17, 95% of MP-ol can be converted to around 78% of acetophenone and guaiacol as desirable aromatic monomeric products and 17.8% of MP-one as a byproduct by using 3% MoS_2_/ZIS-300 after 2 h of visible light irradiation. In the real lignin, the native β-O-4 motif not only feature a benzylic hydroxyl group at the α position (such as PP-ol) but also incorporate a hydroxymethyl group at the β-position.^64−66^ As a lignin model with both key functional groups, PPP-ol was used to investigate the cleavage of C_β_–O bonds to aromatic monomers. As shown in Scheme 3b and Figure S17, 95.7% of PPP-ol is converted to 74.3% of phenol, 17.5% of acetophenone, and 69.5% of acrylophenone as desirable aromatic monomers after 2 h of visible light irradiation. DMP-ol, as a lignin model with both key functional groups and three methoxy groups, is the most similar to the β-O-4 motif in real lignin. As shown in Scheme 3c and Figure S17, 89.8% DMP-ol is converted to 82.7% guaiacol, 14.8% DACE, and 68.9% DPE-one as desirable aromatic monomers. These results confirm the good feasibility of 3% MoS_2_/ZIS-300 monolayer photocatalysts in the cleavage of the C_β_–O bond in different typical β-O-4 lignin models to aromatic monomers. All these lignin model compounds show a slower conversion rate than PP-ol. The results might be attributed to the presence of electron-donating groups (EDG) in lignin model compounds, such as methoxy substituents and C_γ_–OH groups, which increase the electron density of lignin model compounds and decrease the adsorption capability of lignin model compounds on the photocatalyst surfaces, therefore prolonging the reaction time.^52^ In addition, the C_α_–OH groups in lignin model compounds play a crucial role in the cleavage of C_β_–O bonds to aromatic monomers, as evidenced by the lack of reactivity of the PEB lignin model compound after 2 h of visible light irradiation (Scheme 3d).

**Figure 7:**
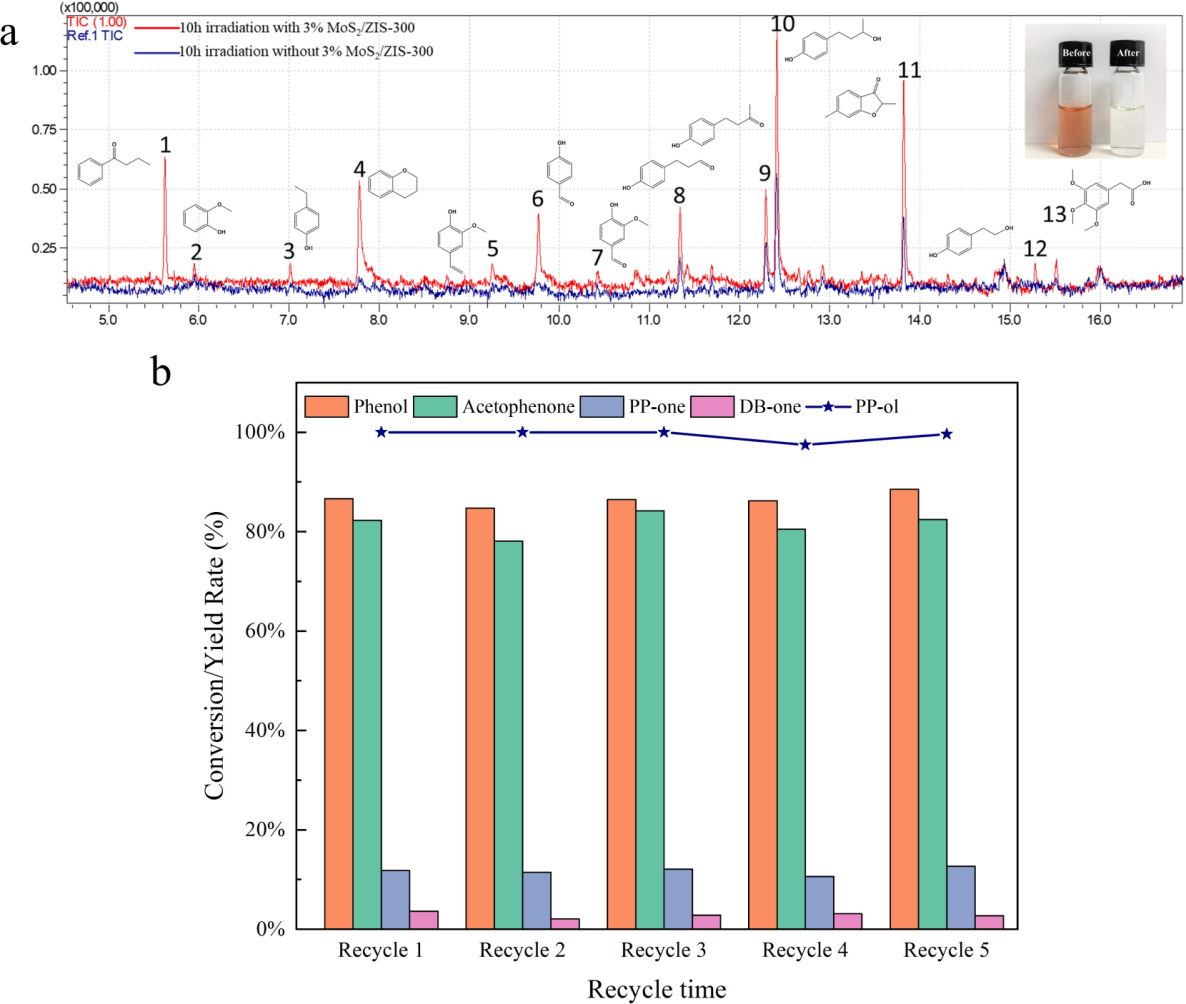
(a) GC-MS spectra for photocatalytically converted products from wood extraction lignin solution. Inset: Solution color before and after the photocatalytic reaction. Reaction conditions: wood extraction powder is 80 mg, CdS-150 is 20 mg, H_2_O and CH_3_CN mixed solution (CH_3_CN/H_2_O (v/v = 2/3)) is 7 mL, Ar is at 1 atm, and visible light power is 0.35 W cm^–2^, 10 h. (b) Conversion rate of PP-ol and yields of products with recycled 3% MoS_2_/ZIS-300 under 1 h of visible light irradiation.

The depolymerization of real lignin extracted from wood sawdust was conducted to assess the potential application of MoS_2_/ZIS-300 monolayer photocatalysts in the production of high-value aromatic monomers. Figures 7a and S18 present the GC-MS results for the products from the fragmentation of real lignin before and after visible light irradiation for 10 h of visible light irradiation. Several new peaks, such as 1 and 3–6 peaks, were observed after 10 h of visible light irradiation. The intensity of existing aromatic monomers peaks also increased 2–5 times after 10 h of visible light irradiation. In addition, the color of the lignin solution changed from tawny to decolorized. All results demonstrate that the 3% MoS_2_/ZIS-300 monolayer is a promising photocatalyst in the efficient fragmentation of real lignin into high-value aromatic monomers.

The photostability of the developed MoS_2_/ZIS-300 monolayer is presented in Figure 7b. After 5 recycling, the recycled 3% MoS_2_/ZIS-300 maintains around 100% conversion of PP-ol and around 85% generation of aromatic monomers after 1 h of visible light irradiation. The XRD patterns for fresh and recycled samples show similar diffraction peaks (Figure S19). The results demonstrate that the 3% MoS_2_/ZIS-300 monolayer has high photostability under photocatalytic conditions.

## 4 Conclusions

The reaction rate of PP-ol and the selectivity of aromatic monomers can be significantly improved by the developed half-unit-cell MoS_2_/ZIS-300 monolayer photocatalysts in this study. The improvement is achieved through enhanced hydrogen transfer efficiency and optimized *OH concentration on the photocatalyst surfaces to improve the C_α_–H bond activation in lignin. The half-unit-cell MoS_2_/ZIS-300 monolayer can improve the migration efficiency of photogenerated electrons from the ZIS-300 monolayer to the MoS_2_ monolayer, and can further enhance the hydrogen transfer efficiency on the surfaces of MoS_2_/ZIS-300 monolayer to promote the production selectivity of aromatic monomers and reduce the generation of DB-one as the C–C coupled byproduct. The MoS_2_/ZIS-300 monolayer photocatalysts show good photostability and feasibility in real lignin conversion experiment.

The *OH on the photocatalyst surfaces can facilitate the C_α_–H bond activation to enhance the conversion rate of PP-ol to aromatic monomers. The conversion rate of PP-ol increases from 65% to 100% through adjusting the ratio of water in the reaction system from 0.2 to 0.6 to optimize the concentration of *OH on the photocatalyst surfaces. The DFT results further demonstrate that the longer C_α_–H bond length at 1.23 Å and the lower BDE of the C_α_–H bond at 1.87 eV on the surfaces of MoS_2_/ZIS-300 with *OH can more readily activate C_α_–H bonds in PP-ol than other two conditions (the MoS_2_/ZIS-300 surfaces without *OH and *H and the MoS_2_/ZIS-300 surfaces with *H). The H_2_O/D_2_O KIE value is around 1.66, which demonstrates that the efficiency of *OH generation on the surfaces of MoS_2_/ZIS-300 is an important step in the C_α_–H bond activation to promote the conversion of PP-ol to aromatic monomers. In summary, PP-ol can be completely converted to 86.6% phenol and 82.3% acetophenone under 1 h of visible light irradiation, which shows the best photocatalytic performance in PP-ol conversion compared to previous studies.

## Supporting Information Available

Experimental details, calibration results, SEM images, XRD patterns, XPS spectra, ICP-OES results, PDOS results, work functions, PL spectra, GC-MS results, ^1^H and ^13^C NMR spectra, and BET results (PDF)
