# Supplementary material for: Engineered Half-Unit-Cell MoS2/ZnIn2S4 Monolayer Photocatalysts and Adsorbed Hydroxyl Radicals-Assisted Activation of Cα–H Bond for Efficient Cβ–O Bond Cleavage in Lignin to Aromatic Monomers
Source: ACS Appl Mater Interfaces. 2024 Aug 31;16(36):47724–40. doi: 10.1021/acsami.4c10515 (PMC11403551; doi:10.1021/acsami.4c10515)
Supplement: Supplementary file 1 — am4c10515_si_001.pdf [file am4c10515_si_001.pdf]

Supporting information.

Engineered Half-unit-cell $MoS_2/ZnIn_2S_4$ Monolayer Photocatalysts and Adsorbed Hydroxyl Radicals Assisted Activation of $C_α$-H Bond for Efficient $C_β$-O Bond Cleavage in Lignin to Aromatic Monomers

Zongyang Yue [a], *Guanchu Lu* [a], *Wenjing Wei* [a], *Yi Huang* [a], *Zheng Chen* [a], *Fergus Dingwall* [a], *Shibo Shao* [a,b,*], *Xianfeng Fan* [a,*]

[a] Institute for Materials and Processes, School of Engineering, The University of Edinburgh, Edinburgh EH9 3BF, U.K.

[b] Petrochemical Research Institute, PetroChina Company Limited, Beijing 102206, China

*X.F.: Tel.: +441316505678; Fax: +441316506551; Email: x.fan@ed.ac.uk;

*S.S.: Tel.: +8615300010615; Email: shaoshibo@petrochina.com.cn.

Photoelectrochemical (PEC) measurements

The photoelectrochemical (PEC) measurements were performed in a standard three-electrode system using an electrochemical workstation (CHI660E, Chenhua, shanghai). The indium tin oxide (ITO) glass with photocatalysts served as the working electrode, with a platinum foil as the counter electrode and an Ag/AgCl electrode as the reference electrode. The electrolyte consisted of 30 mg PP-ol and 0.2 M Na_2ClO_4 in a mixture of 20 mL CH_3CN and 30 mL H_2O. A xenon arc lamp (manufactured by Perfect Light Company) equipped with a PE300BF type light bulb and a 420 nm UV filter was used as light source. For the working electrode, 5 mg of photocatalysts was dispersed in 40 μL ethanol with 5 μL Nafion. The obtained slurry was then evenly spread onto a 3.0 × 1.0 cm^2 conducting ITO glass substrate with an active area of about 1.0 cm^2 and then dried in air.

Alkylation and Regeneration Experiments

The alkylation and regeneration of thiol groups experiments were modified from the method reported in the literature [1]. For the inhibition of thiol groups on the 3% MoS_2/ZIS-300 photocatalyst surface, 50 mg of 3% MoS_2/ZIS-300 and 20 μL of BPTMOS were added into 10 mL of cyclohexane and then stirred at 80 °C for 6 h. After the reaction, the BPTMOS treated 3% MoS_2/ZIS-300 was collected by centrifugation and rinsed with cyclohexane several times before being dried under vacuum at 60 °C for 4 h. As for the regeneration, the inhibition of -SH groups on the surface could be removed by an aqueous solution of NaSH. Specifically, 30 mg of BPTMOS treated 3% MoS_2/ZIS-300 photocatalyst and 100 mg of NaSH were added into 10 mL of deionized water, and then the mixture was stirred at 60 °C for 2 h. After the regeneration, the obtained 3% MoS_2/ZIS-300 was collected by centrifugation. The sample was washed with water several times and then dried in vacuum oven at 60 °C for 4 h.

Measurement of Relative Concentration of *OH Radical Intermediates using PL with Coumarin (Cou)

PL with Cou was used as a molecular probe to evaluate the relative concentration of *OH radical intermediates in the reaction system [2]. Specifically, 0.1 mM Cou was added to the reaction system with varying ratios of water from 0 to 0.8. 3% $MoS_2/ZnIn_2S_4$ photocatalysts

were dispersed by magnetic stirring, and the reactor was sealed tightly after 30 min of argon purge (10 mL min^{-1}). The sealed reactor with 200 rpm of magnetic stirring was illuminated under 1 h of visible light irradiation. The solution was collected by centrifugation and transferred into a cuvette. Photoluminescence (PL) measurements (RF-6000, Shimadzu) were conducted at an excitation wavelength of 335 nm to detect the relative concentration of *OH radical intermediates in different ratios of water.

PL Emission Spectra of 3% MoS_2/ZIS-300 with and without PP-ol

In the photocatalytic reaction system, the interaction between photogenerated charge carriers and PP-ol is a crucial step in the cleavage of the C_β-O bond in PP-ol to aromatic monomers. Therefore, PL measurements were conducted with and without PP-ol to evaluate this interaction. For the experiment, 10 mg 3% MoS_2/ZIS-300 photocatalysts were added to the reaction solvent (CH_3CN/H_2O, v/v = 2/3) either with or without 10 mg of PP-ol. The mixture was then transferred to a cuvette. Photoluminescence (PL) measurements (RF-6000, Shimadzu) were performed at an excitation wavelength of 420 nm to detect the emission intensity of the prepared solution, both with and without PP-ol.

Scavengers Controlled Experiments

In the PP-ol conversion process, the photogenerated holes (h^+) and electrons (e^-), hydroxyl radicals from water oxidation and the formation of C_α radical intermediates through activation of C_α-H bond in PP-ol play critical roles in improving conversion rate to desirable aromatic monomers. To investigate these roles, different scavengers were used to capture C_α radicals, h^+, and e^-. These scavengers include radical scavengers (30 mg of DMPO), C_α radical scavengers (30 mg of TEMPO), hole scavengers (20 mg of Na_2S and 10 mg of Na_2SO_3), hydroxyl radical scavengers (0.1 mM of Cou) and electron scavengers (30 mg of $Na_2S_2O_8$). In detail, 10 mg of PP-ol, 10 mg of 3% MoS_2/ZIS-300, and the specific amount of respective scavengers were added to the reaction solvent (CH_3CN/H_2O, v/v = 2/3). After 1 h of visible light irradiation, the obtained solution was analyzed by GC to calculate the conversion rate of PP-ol and the yields of various products.

Lignin Extraction from Ground Birch Sawdust

Lignin was extracted from ground birch sawdust using a Soxhlet extractor [3,4]. In detail, 10 g of ground birch sawdust was mixed with 160 mL of ethanol, 40 mL of water, and 4 mL of aqueous HCl solution (37%) in a Soxhlet extractor. The extraction process was performed at a temperature of 80 °C. After 20 h of extraction, the extracted lignin was collected by rotary evaporation. The obtained lignin was then washed with water several times and vacuum dried at 60 °C for 2 h.

Theoretical Calculation Details:

Vienna ab-initio simulation package (VASP) was used to conduct the density functional theory (DFT) calculations. The projector augmented wave (PAW) potential and Perdew-Burke-Ernzerhof generalized gradient approximation (PBE-GGA) for exchange-correlation function were applied to describe the ionic cores [5–8]. A plane wave basis set with cutoff energy was conducted at 500 eV. The convergence criterion for total energy was set at 10^{-5} eV and the residual force was 0.02 eV $Å^{-1}$. Partial occupancies of the Kohn−Sham orbitals were allowed using the Gaussian smearing method with a width of 0.10 eV. Electronic energy was considered self-consistent when the energy change of the whole simulated system was smaller than 10^{-7} eV. Geometry optimization was considered convergent when the energy change was smaller than 10^{-6} eV. Grimme's DFT-D3 methodology was used to describe the dispersion interactions among all the atoms.

Bulk $ZnIn_2S_4$ and MoS_2 were calculated using their primitive cells, incorporating k-point meshes of 5 × 5 × 5 and 16 × 16 × 16, respectively, to ensure accurate Brillouin zone sampling. To simulate the $ZnIn_2S_4$ monolayer accurately and avoid interactions from periodic images, a vacuum region of 15 Å was introduced. For the heterojunction model comprising a half-unit-cell $MoS_2/ZnIn_2S_4$ monolayer, both components were simulated with a 3 × 3 × 3 k-point mesh and separated by a 15 Å vacuum region, ensuring minimal interaction across the periodic boundary in the two-dimensional monolayer system.

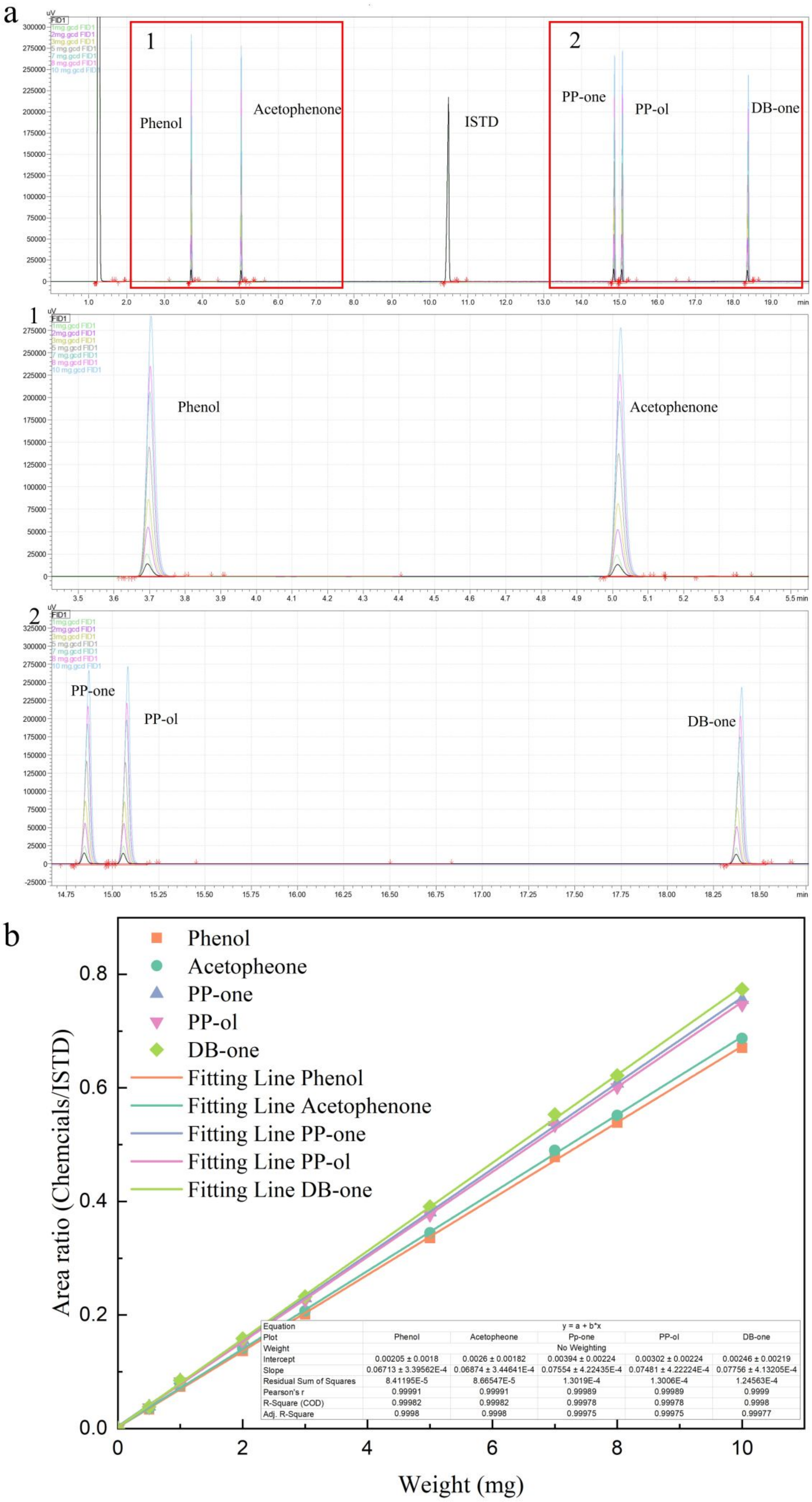

Equation	y = a + b*x				
Plot	Phenol	Acetopheone	Pp-one	PP-ol	DB-one
Weight	No Weighting				
Intercept	0.00205 ± 0.0018	0.0026 ± 0.00182	0.00394 ± 0.00224	0.00302 ± 0.00224	0.00246 ± 0.00219
Slope	0.06713 ± 3.39562E-4	0.06874 ± 3.44641E-4	0.07554 ± 4.22435E-4	0.07481 ± 4.22224E-4	0.07756 ± 4.13205E-4
Residual Sum of Squares	8.41195E-5	8.66547E-5	1.3019E-4	1.3006E-4	1.24563E-4
Pearson's r	0.99991	0.99991	0.99989	0.99989	0.9999
R-Square (COD)	0.99982	0.99982	0.99978	0.99978	0.9998
Adj. R-Square	0.9998	0.9998	0.99975	0.99975	0.99977

Figure S1. (a) GC spectra of various weights of chemicals (0.5 mg, 1 mg, 2 mg, 3 mg, 5 mg, 7 mg, 8 mg, 10 mg) for calibration to calculate the conversion rate of PP-ol and the selectivity/yield of all generated products. The chemicals include phenol, acetophenone, PP-ol, PP-one, and DB-one. (b) Calibration fitting curves for these chemicals based on the GC results.

The quantitative analysis of PP-ol and products was determined using the internal standard method, which helps minimize errors during the measurement of reaction solutions. Specifically, various weights of substrates were added to 5 mL of reaction solution along with 8 mg of methylparaben, used as the internal standard (ISTD), to prepare standard samples. These samples were then tested using GC equipment (**Figure S1a**). The following equation based on the calibration curve was used to determine the unknown weight of compounds:

$A/A_{ISTD} = a \times m_{chemcial} + b$

Where A is the peak area of a compound, A_{ISTD} is the peak area of internal standard, $m_{chemcials}$ is the weight of a compound. a is the slope compensation factor and b is the constant compensation factor, which is determined from the calibration process.

Calibration equations were obtained for different chemical compounds as shown in **Figure S1b** and the inset table in **Figure S1b**. In these equations, y represents A/A_{ISTD}, and x represents $m_{chemcial}$. The errors (R^2) of the fitting equations are all above 0.999. Based on the calculated weights of different chemical compounds, the conversion rate of PP-ol and selectivity/yield of the products can be accurately determined.

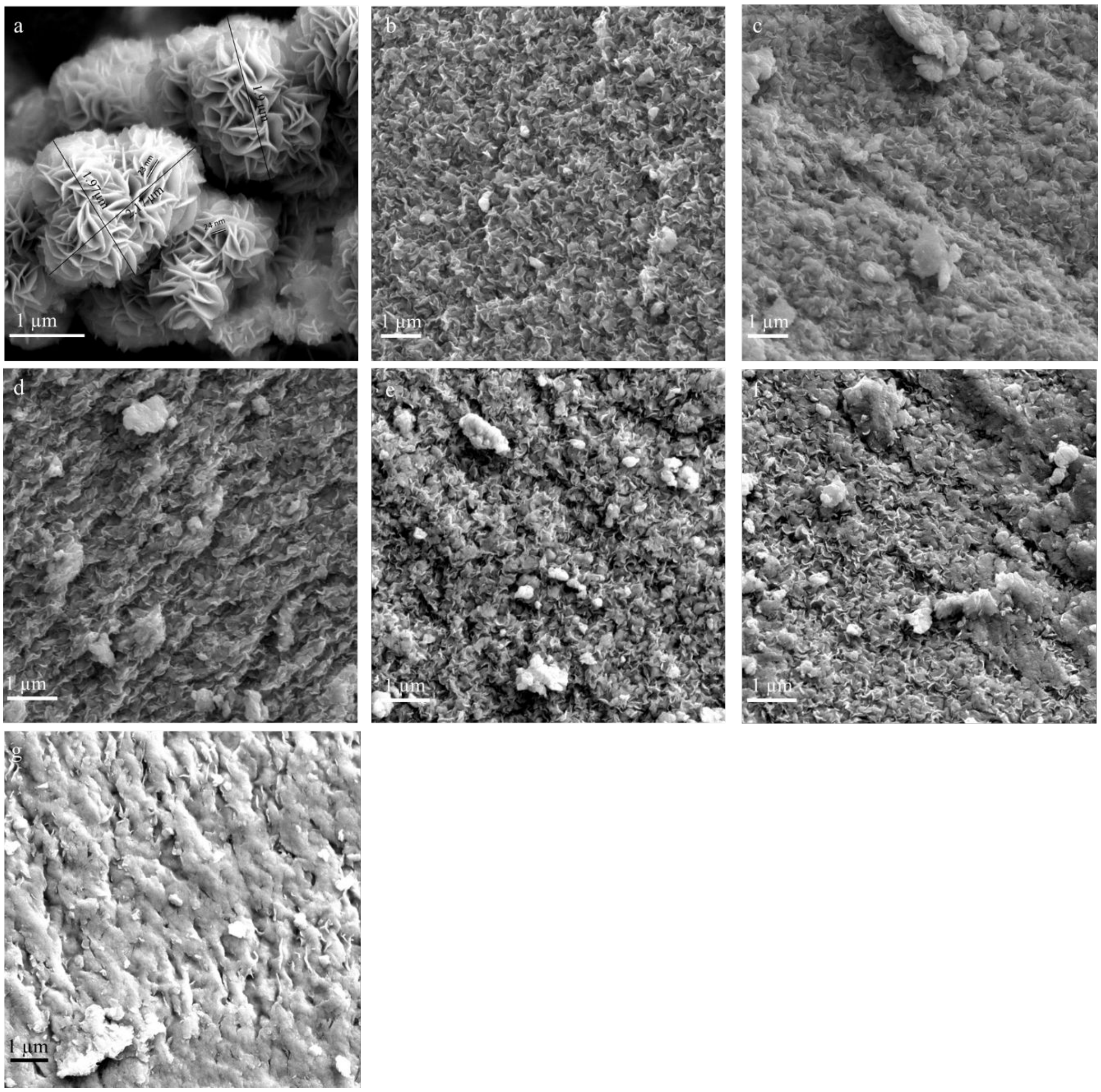

Figure S2. SEM images of (a) ZIS-0, (b) ZIS, (c) 0.5% MoS_2/ZIS-300, (d) 1.5% MoS_2/ZIS-300, (e) 3% MoS_2/ZIS-300, (f) 5% MoS_2/ZIS-300, (g) 7.5% MoS_2/ZIS-300.

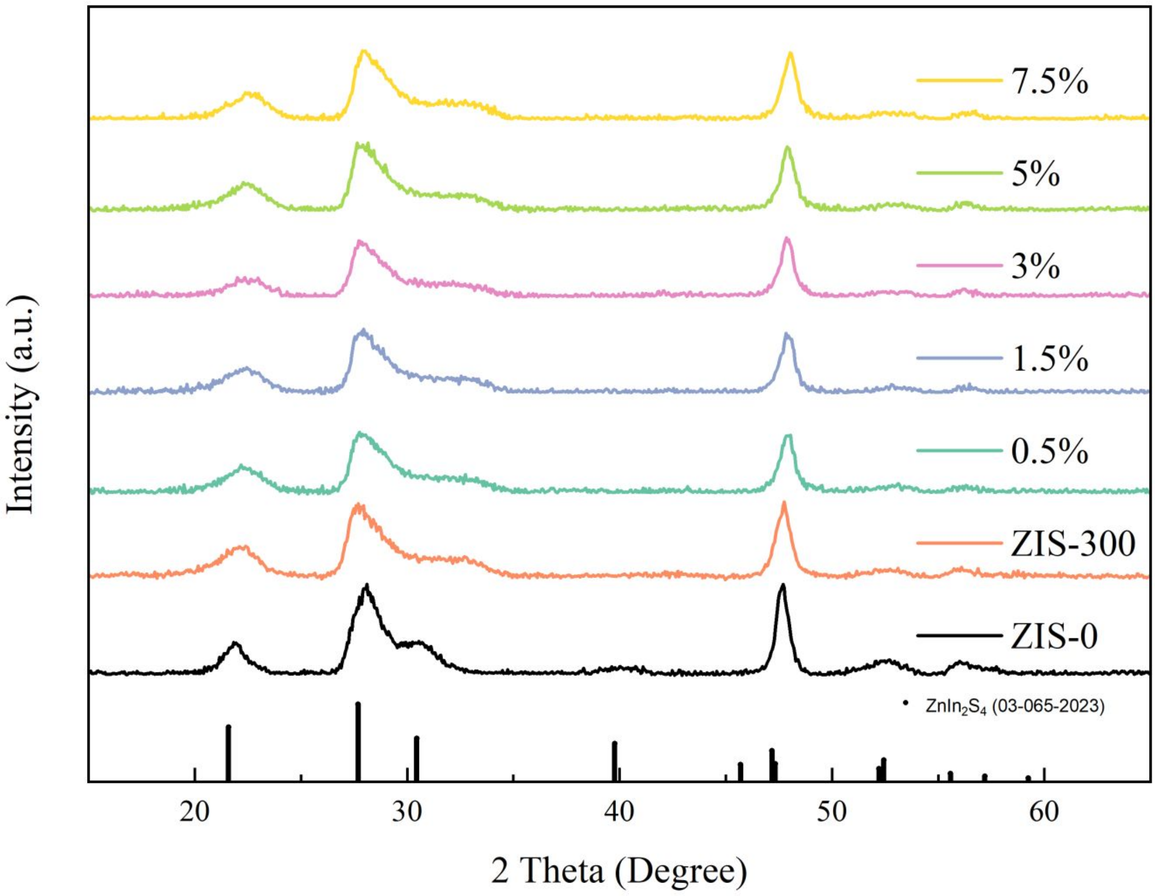

Figure S3. (a) XRD patterns of ZIS-0, ZIS-300, 0.5% MoS_2/ZIS-300, 1.5% MoS_2/ZIS-300, 3% MoS_2/ZIS-300, 5% MoS_2/ZIS-300 and 7.5% MoS_2/ZIS-300

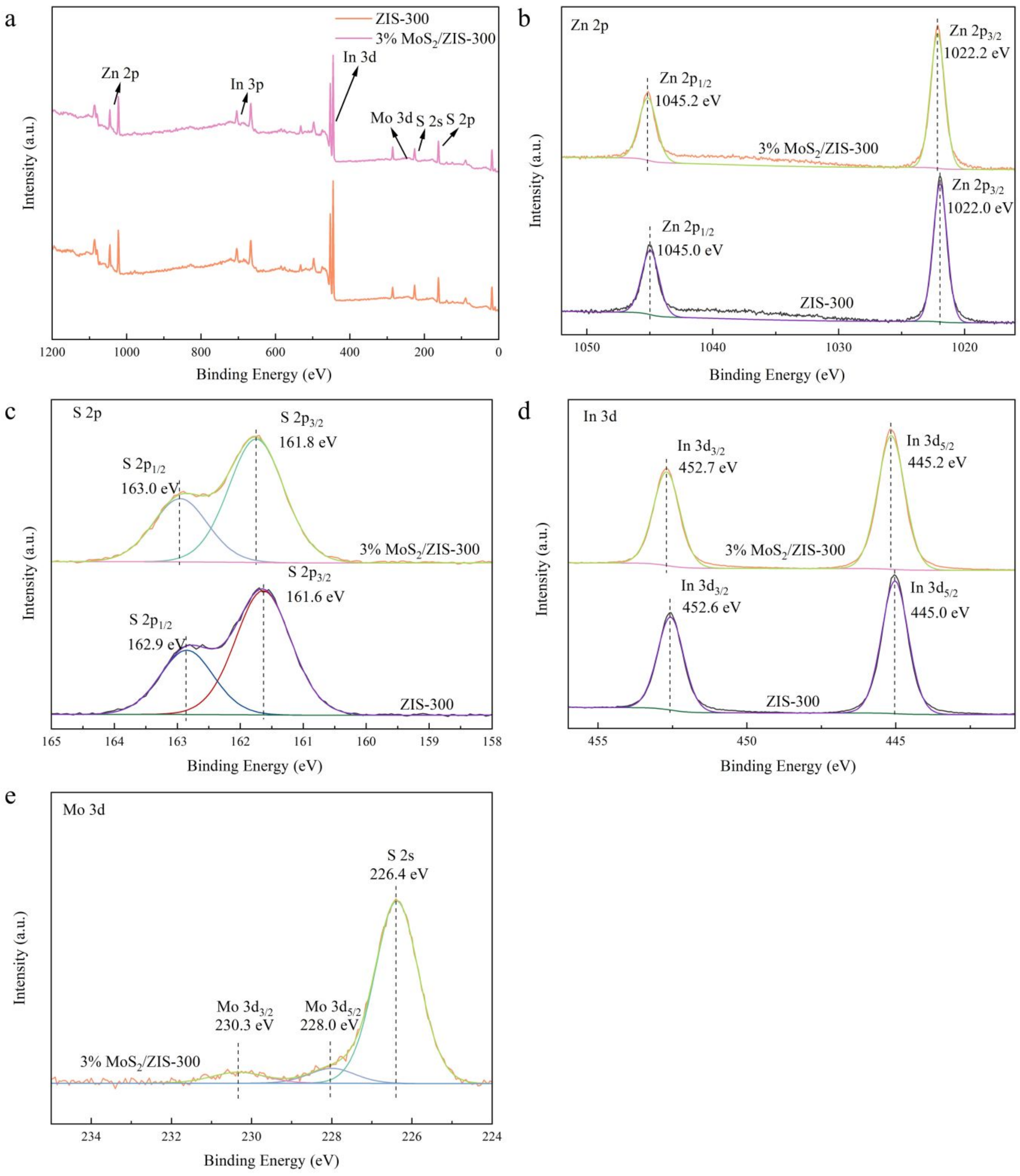

Figure S4. (a) Full XPS spectrum of ZIS-300 and 3% MoS_2/ZIS-300; High-resolution XPS spectra of ZIS-300 and 3% MoS_2/ZIS-300: (b) Zn 2p states, (c) S 2p states, (d) In 3d states. (e) High-resolution XPS spectra of Mo 3d states in 3% MoS_2/ZIS-300.

Table S1. The results of elemental analysis by ICP-OES.

Photocatalysts	Mo/Zn (atomic ratio)	In/Zn (atomic ratio)
ZIS-300	-	2.18
0.5% MoS_2/ZIS-300	0.26%	2.21
1.5% MoS_2/ZIS-300	0.29%	2.14
3% MoS_2/ZIS-300	0.39%	2.19
5% MoS_2/ZIS-300	0.43%	2.24
7.5% MoS_2/ZIS-300	0.44%	2.15

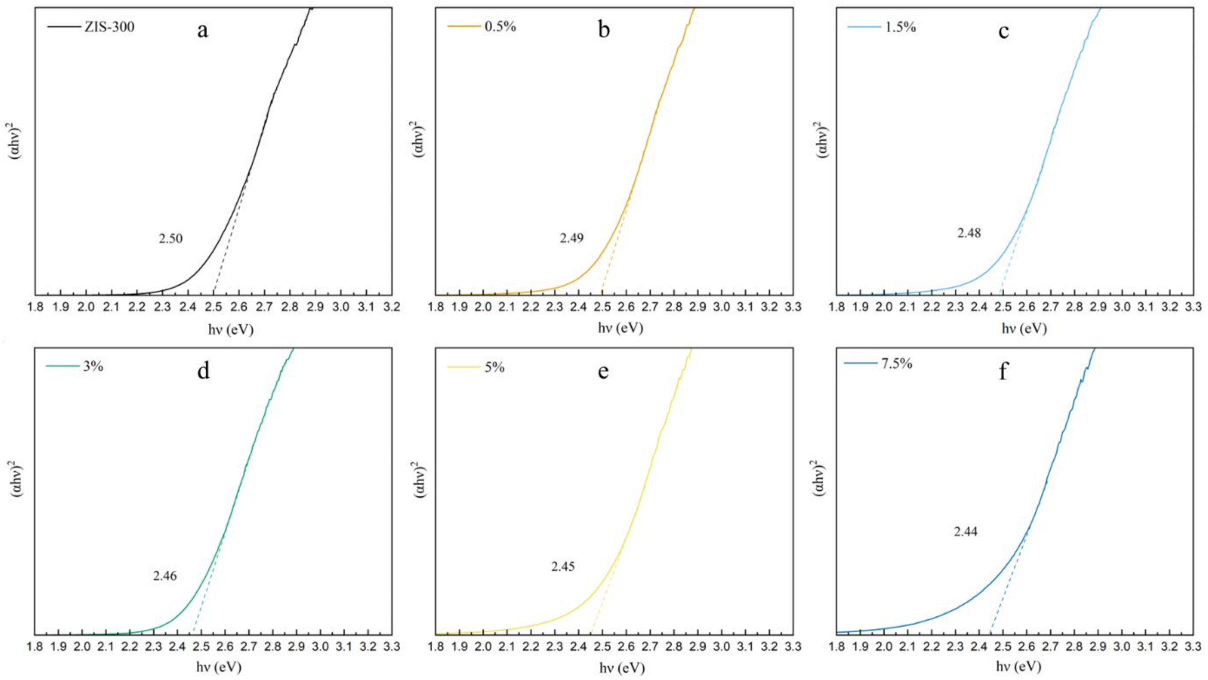

Figure S5. $(\alpha h\nu)^2$ plots versus hν curves for x% MoS_2/ZIS-300 nanoparticles: (a) ZIS-300, (b) 0.5% MoS_2/ZIS-300, (c) 1% MoS_2/ZIS-300, (d) 3% MoS_2/ZIS-300, (e) 5% MoS_2/ZIS-300 and (f) 7.5% MoS_2/ZIS-300.

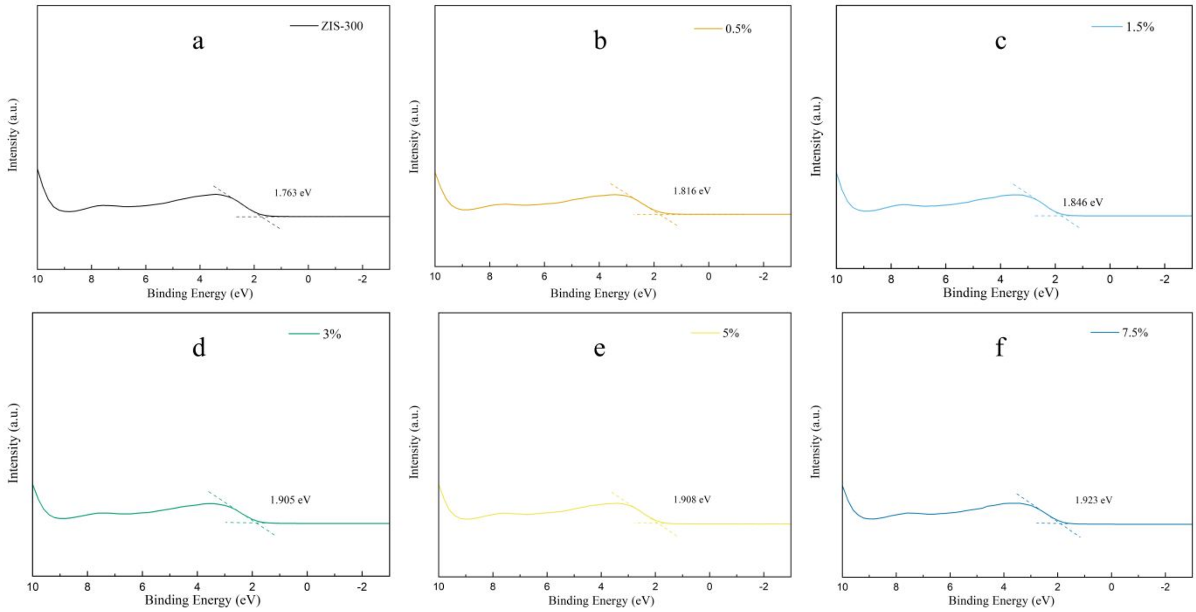

Figure S6. Valence-band XPS spectra for x% MoS_2/ZIS-300 nanoparticles: (a) ZIS-300, (b) 0.5% MoS_2/ZIS-300, (c) 1% MoS_2/ZIS-300, (d) 3% MoS_2/ZIS-300, (e) 5% MoS_2/ZIS-300 and (f) 7.5% MoS_2/ZIS-300.

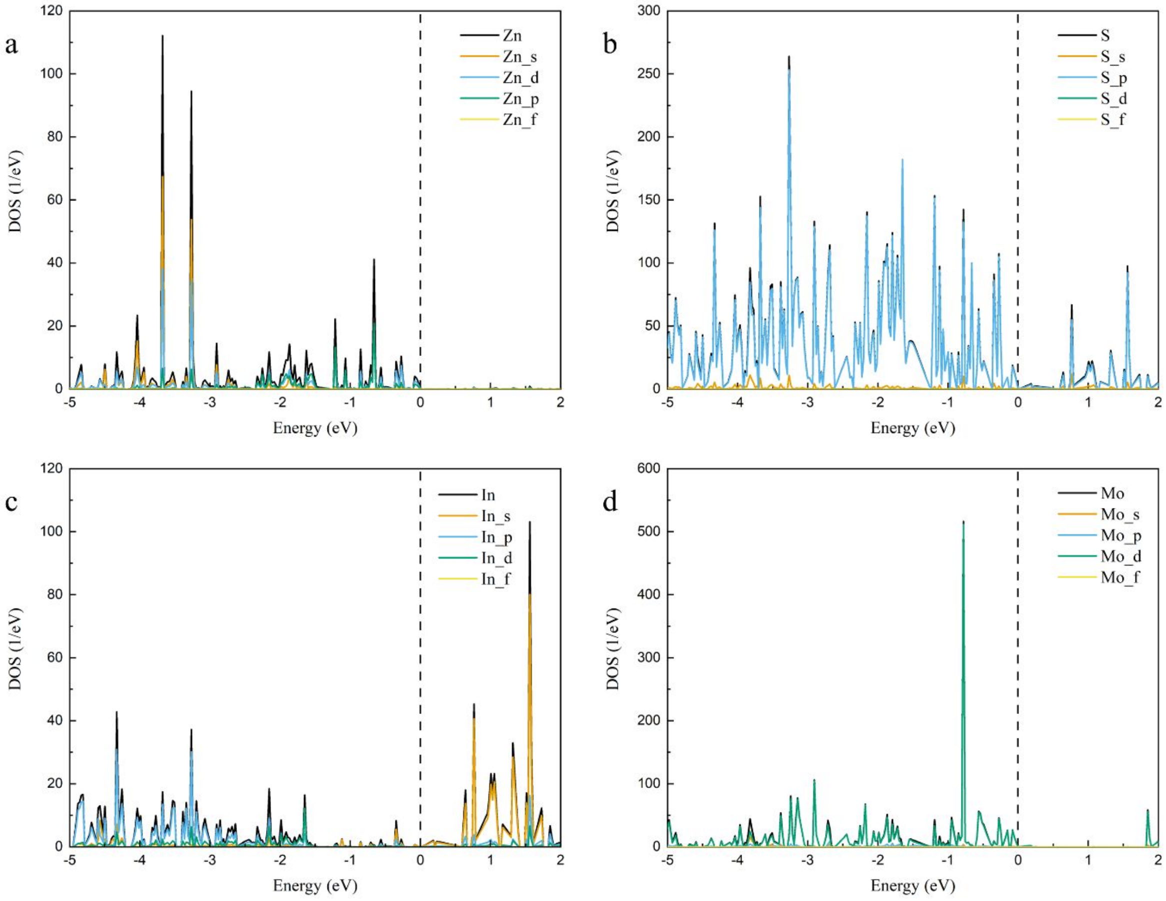

Figure S7. PDOS of (a) Zn; (b) S; (c) In; (d) Mo in half-unit-cell MoS_2/ZIS-300 monolayer.

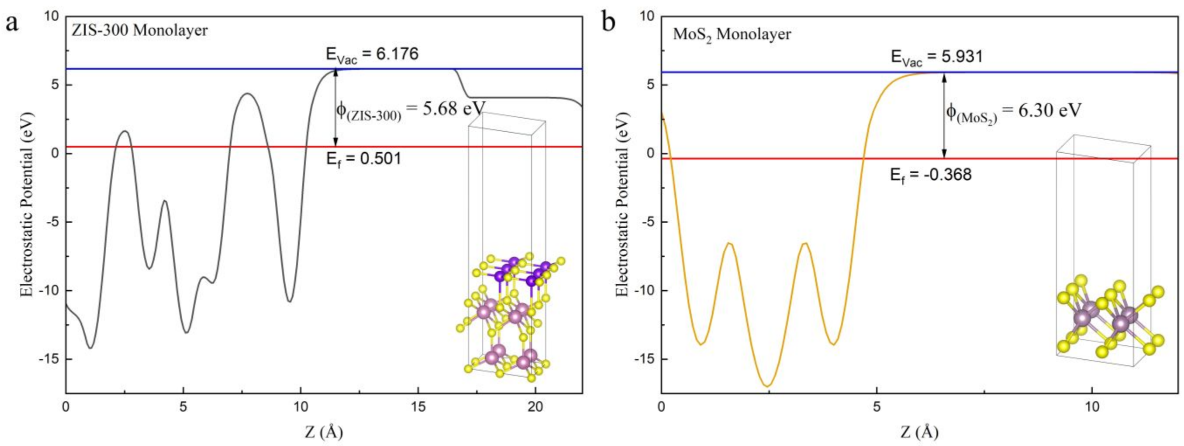

Figure S8. Calculated average potential profile along the Z axis of (a) ZIS-300 monolayer and (d) MoS_2 monolayer.

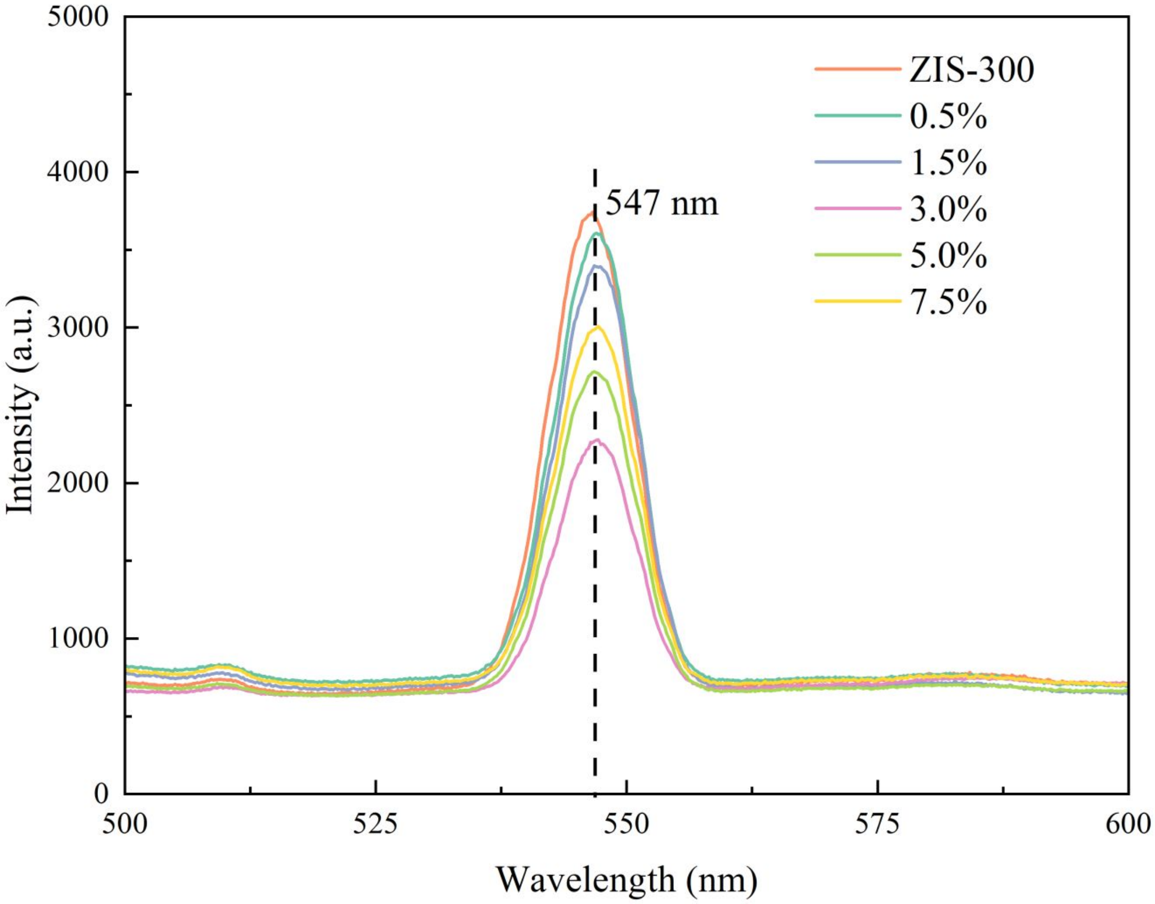

Figure S9. Powdery PL spectra of x% MoS_2/ZIS-300 ($\lambda_{excitation}$ = 400 nm).

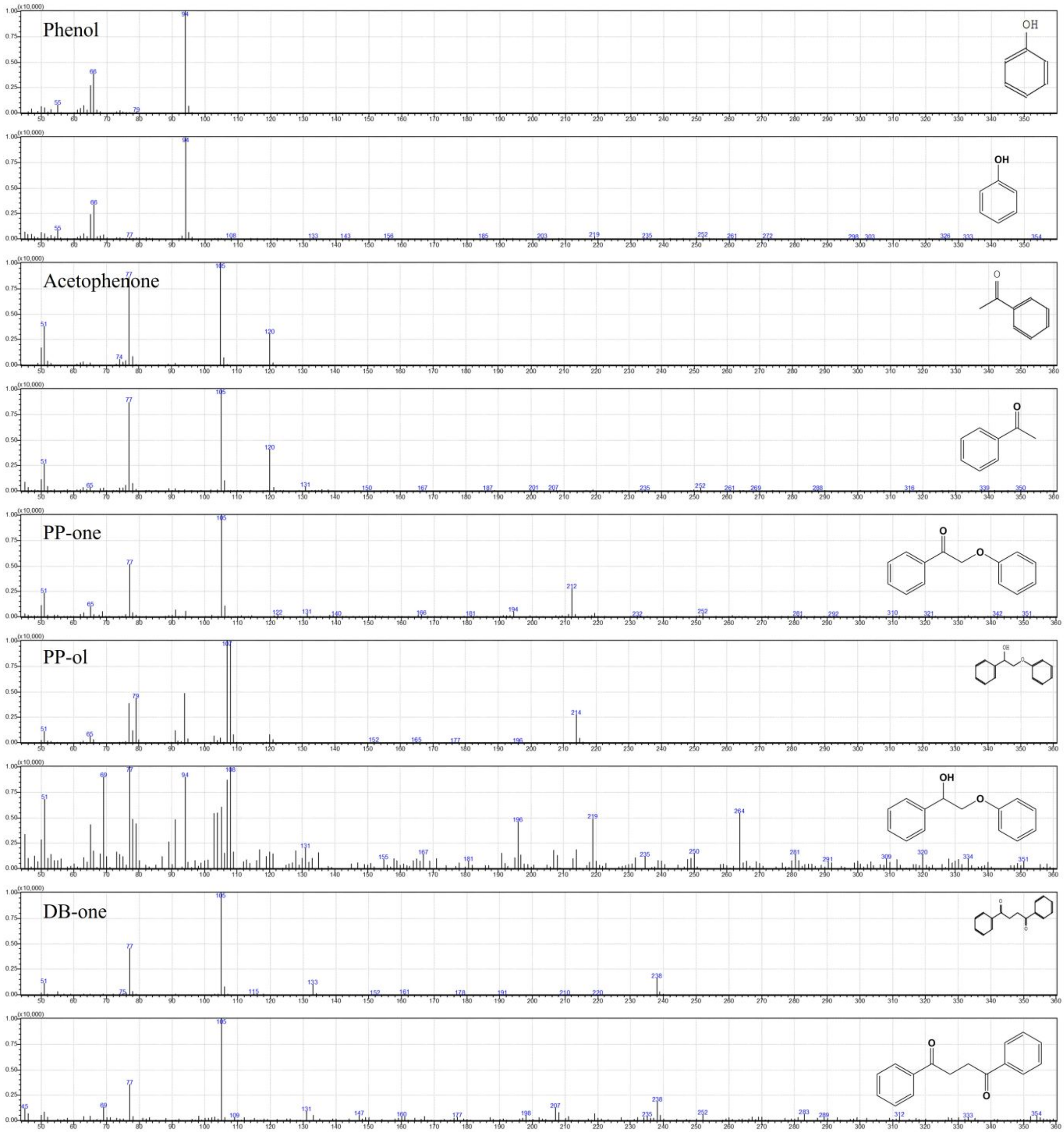

Figure S10. The standard (upper) and detected (bottom) mass spectra of generated compounds after the photocatalytic reactions. Typical reaction condition: lignin model compound PP-ol is 10 mg, photocatalyst is 10 mg, solvent (CH_3CN/H_2O (v/v = 2/3)) is 5 mL, Ar is at 1 atm, visible light power is 0.35 W cm^{-2}, 1h.

GC-MS analysis was conducted to qualify the generated products from photocatalytic conversion of PP-ol. The results were then compared with the standard mass spectra of these products in the library. As shown in **Figure S10**, both the standard and detected mass spectra of phenol, acetophenone, PP-ol, and DB-one from GC-MS analysis were presented to

confirm the generated products were phenol, acetophenone, and DB-one. For PP-one, the standard mass spectra were not available in the GC-MS library, so we compared it with previously published results [9] and confirmed the generation of PP-one is one of byproducts in the photocatalytic conversion of PP-ol.

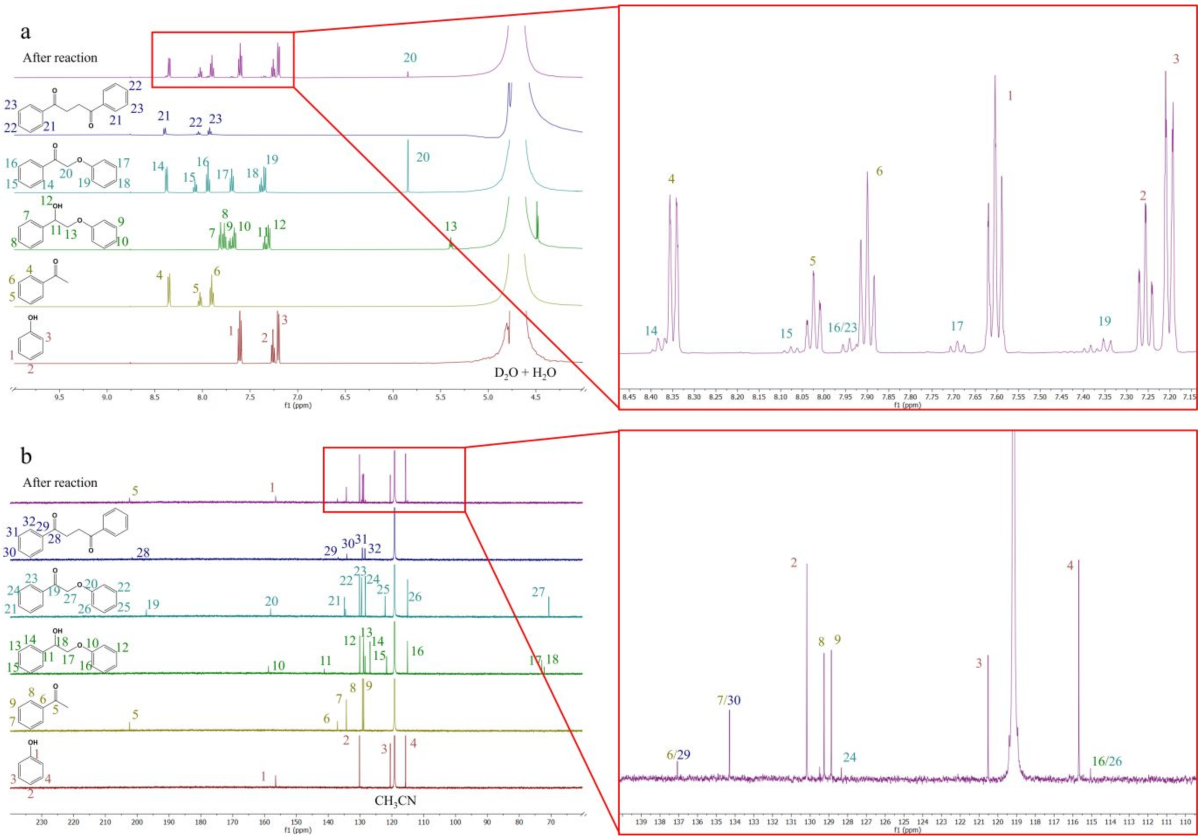

Figure S11. The ^{1}H and ^{13}C NMR spectra for standard chemicals and the reaction solution. For NMR analysis of standard chemicals, chemical is 10 mg, solvent (CH_3CN/H_2O (v/v = 2/3)) is 2.5 mL, D_2O is 2.5 mL; for NMR analysis of reaction solution, 2.5 mL reaction solution is mixed with 2.5 mL D_2O. Typical reaction condition: lignin model compound PP-ol is 10 mg, photocatalyst is 10 mg, solvent (CH_3CN/H_2O (v/v = 2/3)) is 5 mL, Ar is at 1 atm, visible light power is 0.35 W cm^{-2}, 1h.

The ^{1}H and ^{13}C NMR analyses were conducted to further identify the generated products from conversion of PP-ol. The products in reaction solution were identified through comparing ^{1}H and ^{13}C NMR spectra of the reaction products with the standard compounds. As shown in **Figure S11**, all peaks in both ^{1}H and ^{13}C NMR spectra of the standard compounds were identified and labelled, and their corresponding chemical compounds clearly indicated in the diagram. All peaks in both ^{1}H and ^{13}C NMR spectra of the reaction solution were also labelled and compared with the peaks of standard compounds to determine the specific products generated after 1 h of visible light irradiation. Both ^{1}H and ^{13}C NMR spectra of the reaction solution primarily show peaks for acetophenone and phenol, with smaller peaks for PP-one and DB-one. The results indicate that the main products of the

reaction are acetophenone and phenol, with minor amounts of PP-one and DB-one after 1 h of visible light irradiation.

Table S2. Photocatalytic performance of heterogenous catalysts for PP-ol conversion in literature.

Ref	Catalyst	Reaction Condition						Conversion	Selectivity			
		Solvent	PP-ol amount	Temp	Atmosphere	Reaction time	Light Source	PP-ol	Phenol	Acetophenone	PP-one	DB-one
10	$ZnIn_2S_4$ (5 mg)	1 mL CH_3CN	0.10 mmol	42 °C	N_2	4 h	9.6 W blue LEDs (455 nm),	>99%	90%	83%	6%	2%
1	$Zn_4In_2S_7$ (10 mg)	5 mL CH_3CN/H_2O (1:1 v/v)	0.10 mmol	-	N_2	4 h	Xe lamp (400–780 nm), 0.6 W/cm^2	99%	82%	86%	9.6%	-
3	Ni/CdS (20 mg)	10 mL CH_3CN/0.1 M KOH (2:8, v/v)	0.10 mmol	-	N_2	2 h	8 W Blue LED (440–460 nm)	~100%	~90%	~90%	-	-
11	SL-Fe_3O_4/TiO_2 (100mg)	50 mL methanol, 0.5 mL 30% H_2O_2	0.5 mmol	40 °C	O_2	12 h	-	94.3%	3.0%	3.5%	-	-
12	ZIF-8-NH_2@Bi/Bi_2MoO_6 (10 mg)	10 mL CH_3CN/H_2O (1:1, v/v)	10 mL of PP-ol (0.05 mmol/L)	20-25 °C	Air	6 h	300 W xenon lamp (λ > 400 nm)	93%	57%	48%	34%	-
11	SL-Fe_3O_4/TiO_2 (100mg)	50 mL methanol, 0.5 mL 30% H_2O_2	0.5 mmol	40 ° C	O_2	12 h	-	94.3%	3.0%	3.5%	-	-
12	ZIF-8-NH_2@Bi/Bi_2MoO_6 (10 mg)	10 mL CH_3CN/H_2O (1:1, v/v)	10 mL of PP-ol (0.05 mmol/L)	20-25 ° C	Air	6 h	300 W xenon lamp (λ > 400 nm)	93%	57%	48%	34%	-
This work	3% MoS_2/ZIS-300 (10mg)	5 mL CH_3CN/H_2O (3:7, v/v)	10 mg	20 °C	Ar	1 h	Xe lamp, (420–780 nm, 0.35W/cm^2)	100%	86.6%	82.3%	3.6%	3.1%

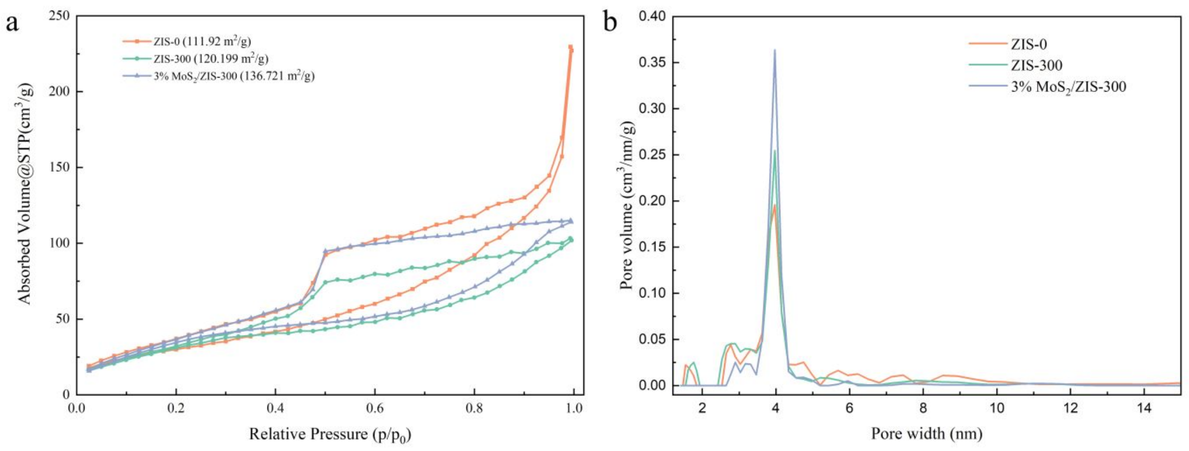

Figure S12. (a) Nitrogen adsorption/desorption isotherms (77 K) with BET specific surface areas and (b) pore size distribution curves of ZIS-0, ZIS-300 and 3% MoS_2/ZIS-300.

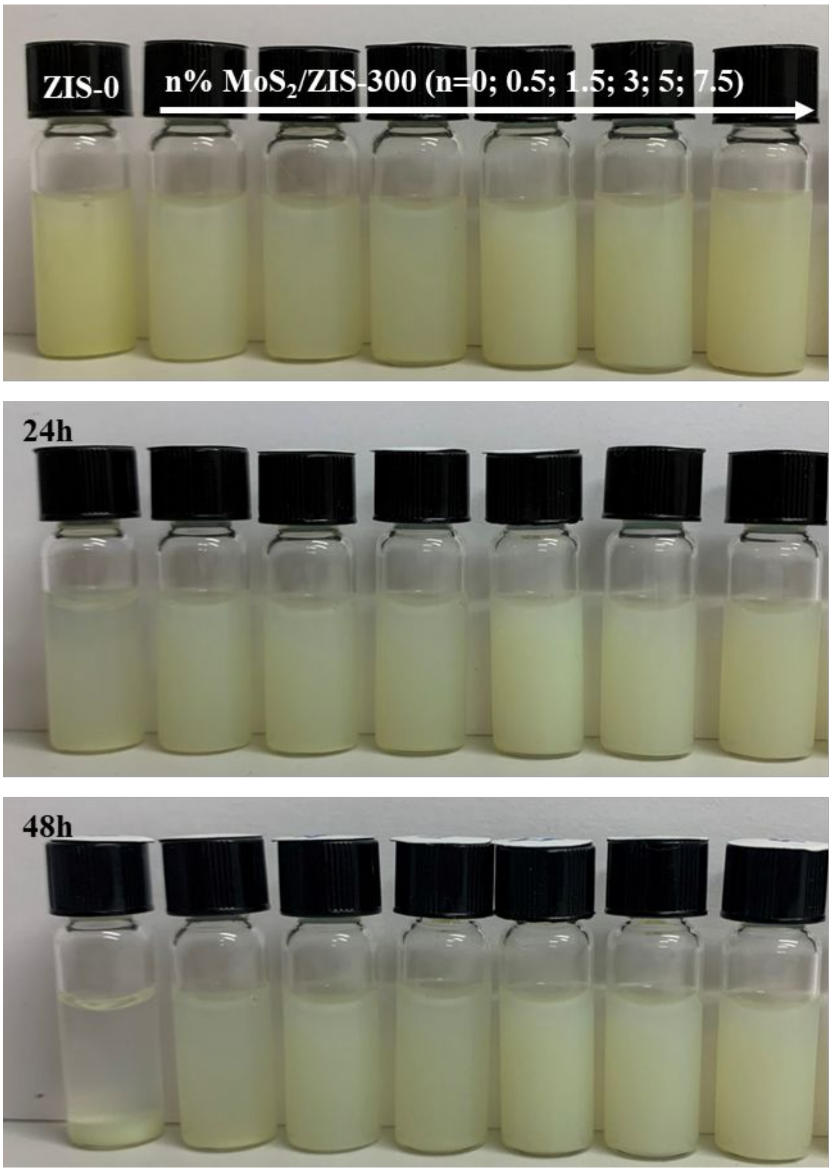

Figure S13. The dispersibility of x% MoS_2/ZIS-300 nanoparticles in the reaction system.

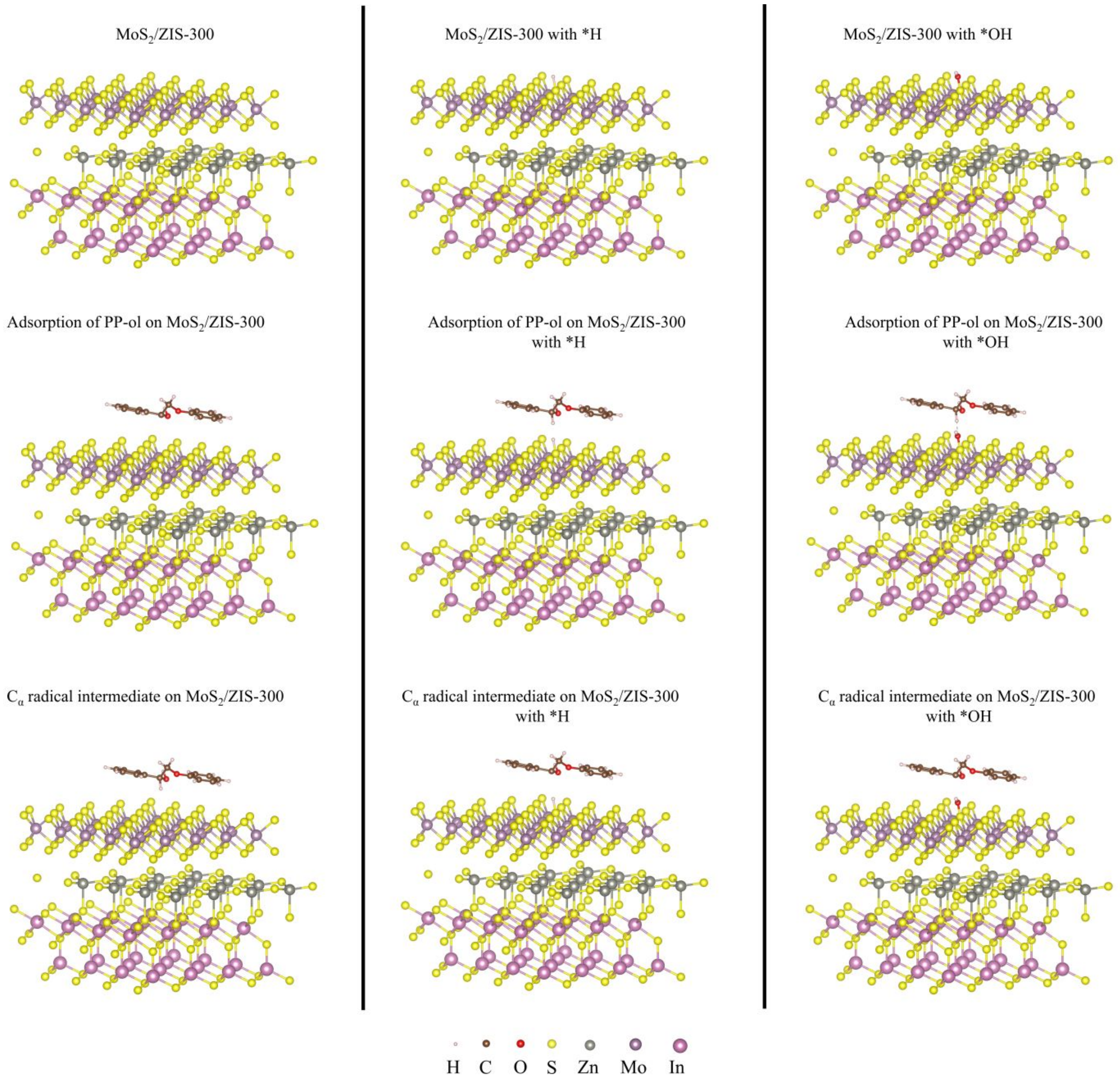

Figure S14. The calculated structure models for adsorption of PP-ol and adsorption of Cα Radical Intermediate on MoS2/ZIS-300 surfaces with and without *H or *OH.

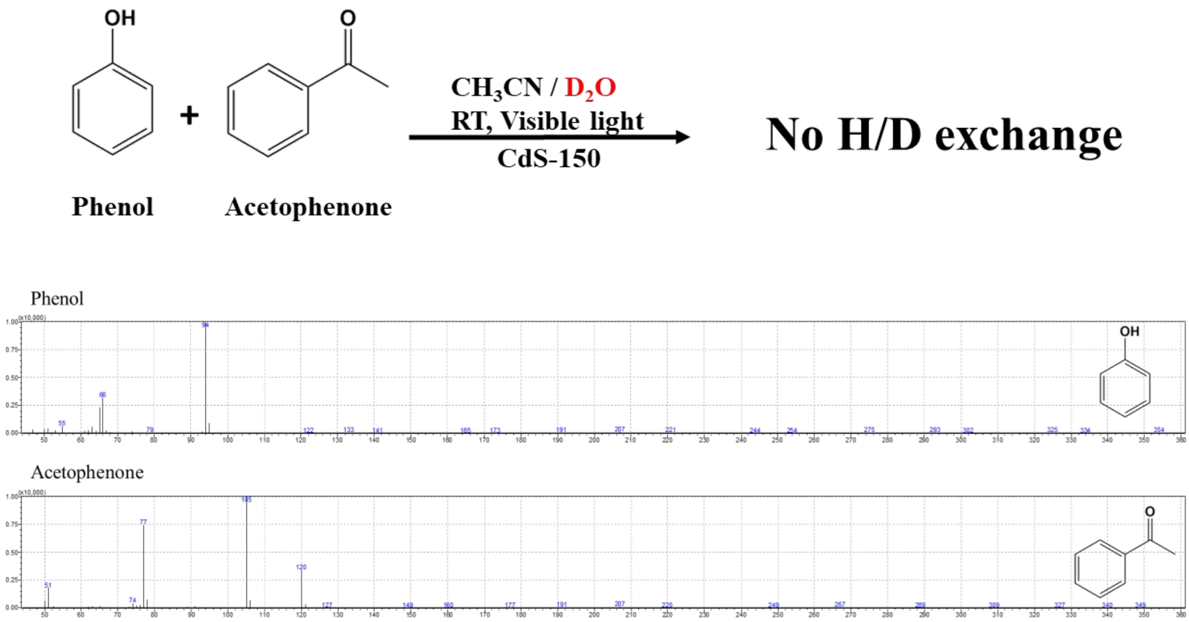

Figure S15. Phenol and acetophenone in D_2O reaction system for H/D exchange. Reaction condition: phenol is 10 mg, acetophenone is 10 mg, 3% MoS_2/ZIS-300 is 10 mg, solvent (CH_3CN/D_2O (v/v = 2/3)) is 5 mL, Ar is at 1 atm, visible light power is 0.35 W cm^{-2}, 1 h.

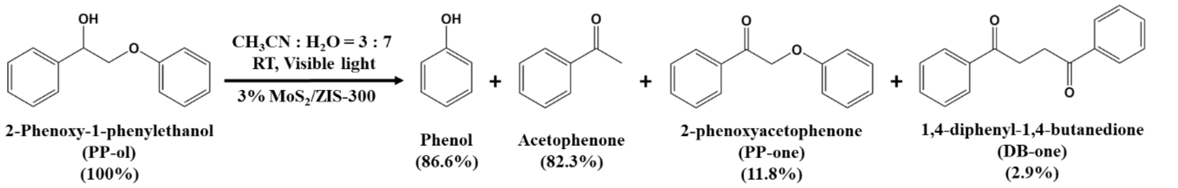
OH
O
CH$_3$CN : H$_2$O = 3 : 7
RT, Visible light
3% MoS$_2$/ZIS-300
2-Phenoxy-1-phenylethanol
(PP-ol)
(100%)
Phenol
(86.6%)
Acetophenone
(82.3%)
2-phenoxyacetophenone
(PP-one)
(11.8%)
1,4-diphenyl-1,4-butanedione
(DB-one)
(2.9%)

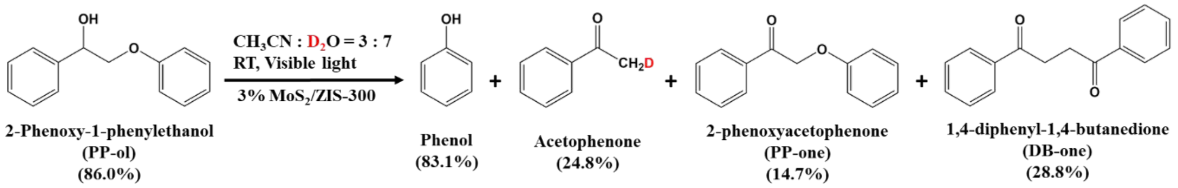
OH
O
CH$_3$CN : D$_2$O = 3 : 7
RT, Visible light
3% MoS$_2$/ZIS-300
CH$_2$D
2-Phenoxy-1-phenylethanol
(PP-ol)
(86.0%)
Phenol
(83.1%)
Acetophenone
(24.8%)
2-phenoxyacetophenone
(PP-one)
(14.7%)
1,4-diphenyl-1,4-butanedione
(DB-one)
(28.8%)

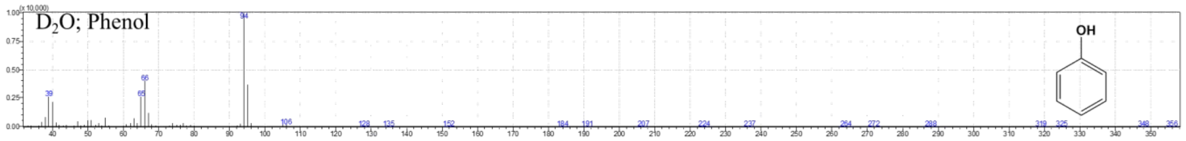
D$_2$O; Phenol
OH

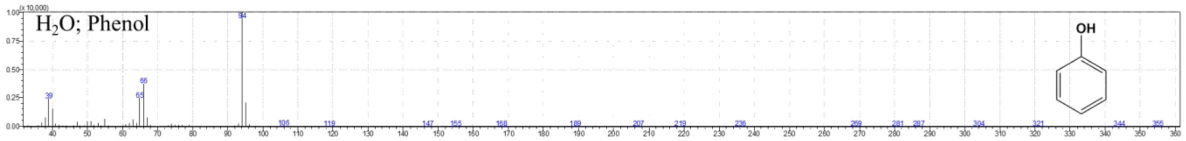
H$_2$O; Phenol
OH

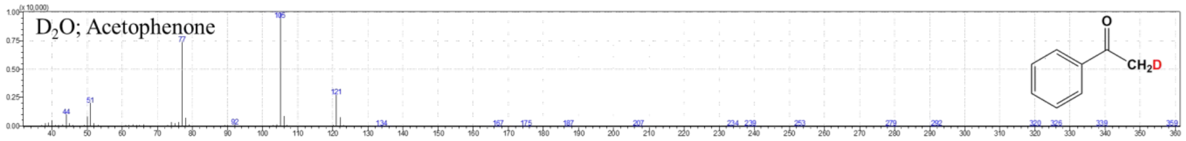
D$_2$O; Acetophenone
O
CH$_2$D

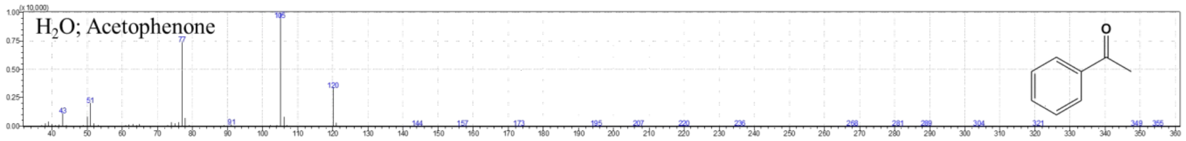
H$_2$O; Acetophenone
O

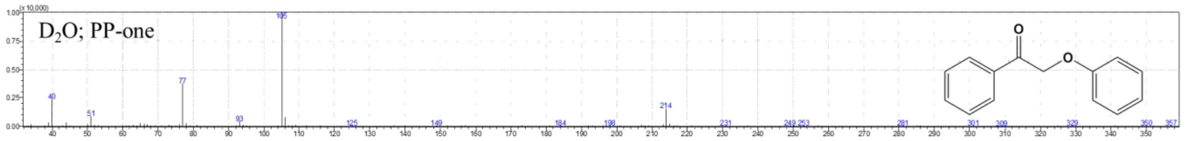
D$_2$O; PP-one
O

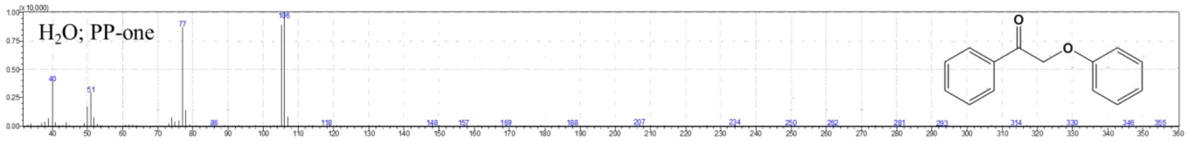
H$_2$O; PP-one
O

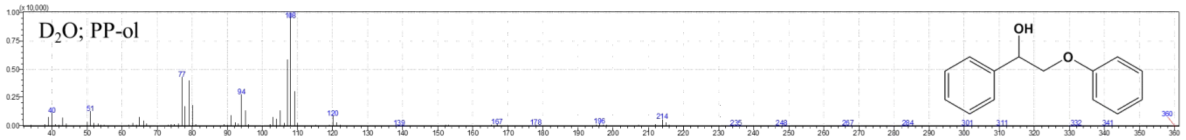
D$_2$O; PP-ol
OH
O

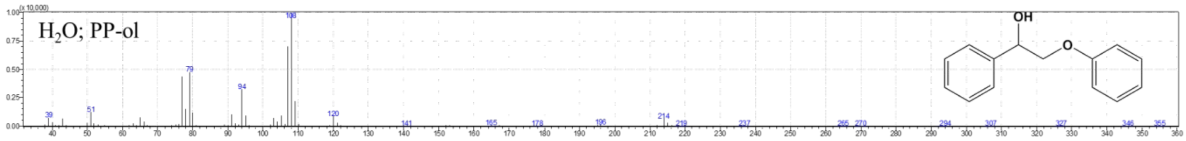
H$_2$O; PP-ol
OH
O

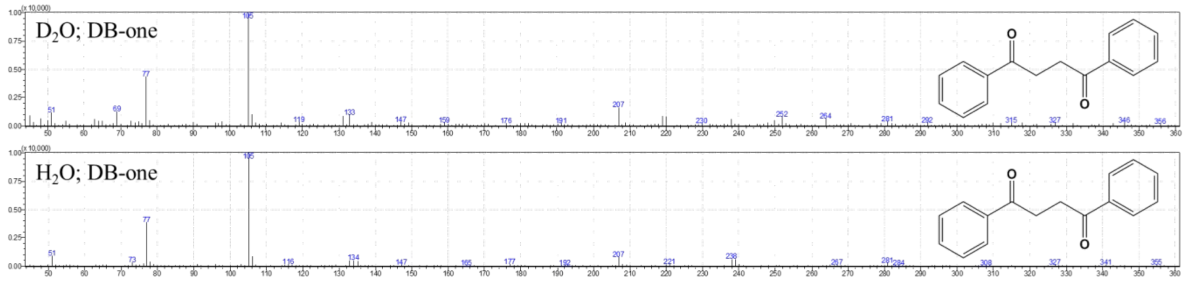

Figure S16. The detected mass spectra of the fragmented PP-ol to acetophenone, phenol and PP-one in H_2O and D_2O solvent conditions. Reaction condition: lignin model compound PP-ol is 10 mg, 3% MoS_2/ZIS-300 is 10 mg, solvent ($CH_3CN/H(D)_2O$ (v/v = 2/3)) is 5 mL, Ar is at 1 atm, visible light power is 0.35 W cm^{-2}, 1 h.

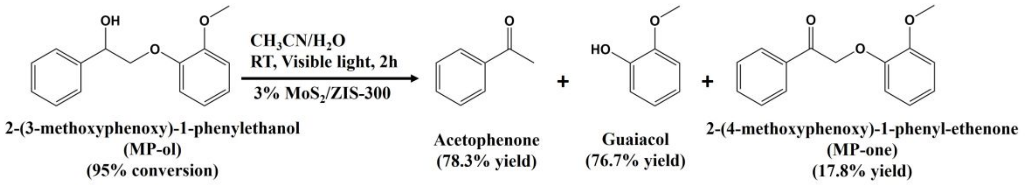
CH3CN/H2O
RT, Visible light, 2h
3% MoS2/ZIS-300
2-(3-methoxyphenoxy)-1-phenylethanol
(MP-ol)
(95% conversion)
Acetophenone
(78.3% yield)
Guaiacol
(76.7% yield)
2-(4-methoxyphenoxy)-1-phenyl-ethenone
(MP-one)
(17.8% yield)

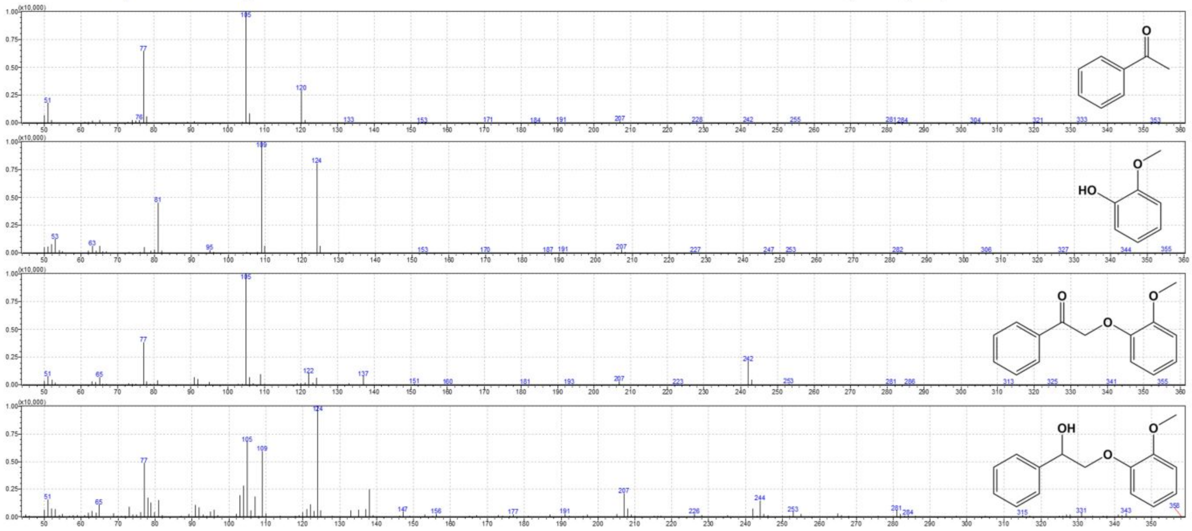

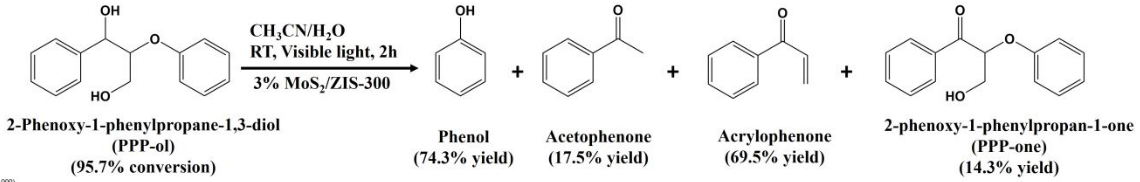
CH3CN/H2O
RT, Visible light, 2h
3% MoS2/ZIS-300
2-Phenoxy-1-phenylpropane-1,3-diol
(PPP-ol)
(95.7% conversion)
Phenol
(74.3% yield)
Acetophenone
(17.5% yield)
Acrylophenone
(69.5% yield)
2-phenoxy-1-phenylpropan-1-one
(PPP-one)
(14.3% yield)

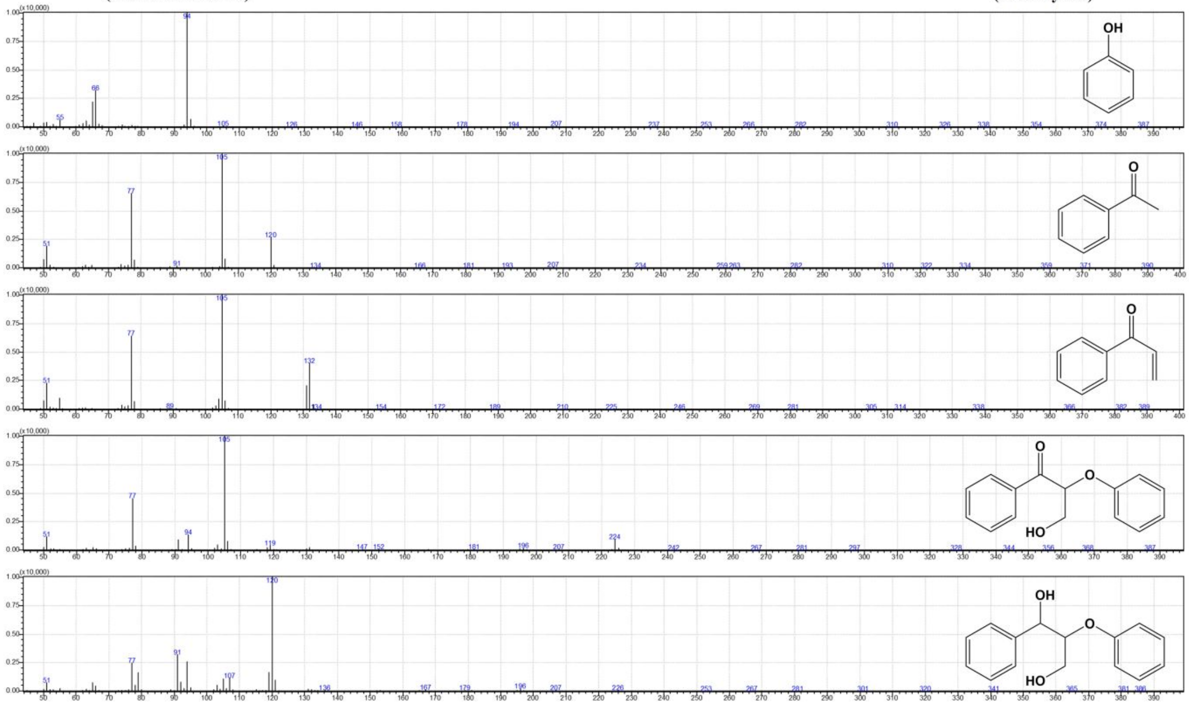

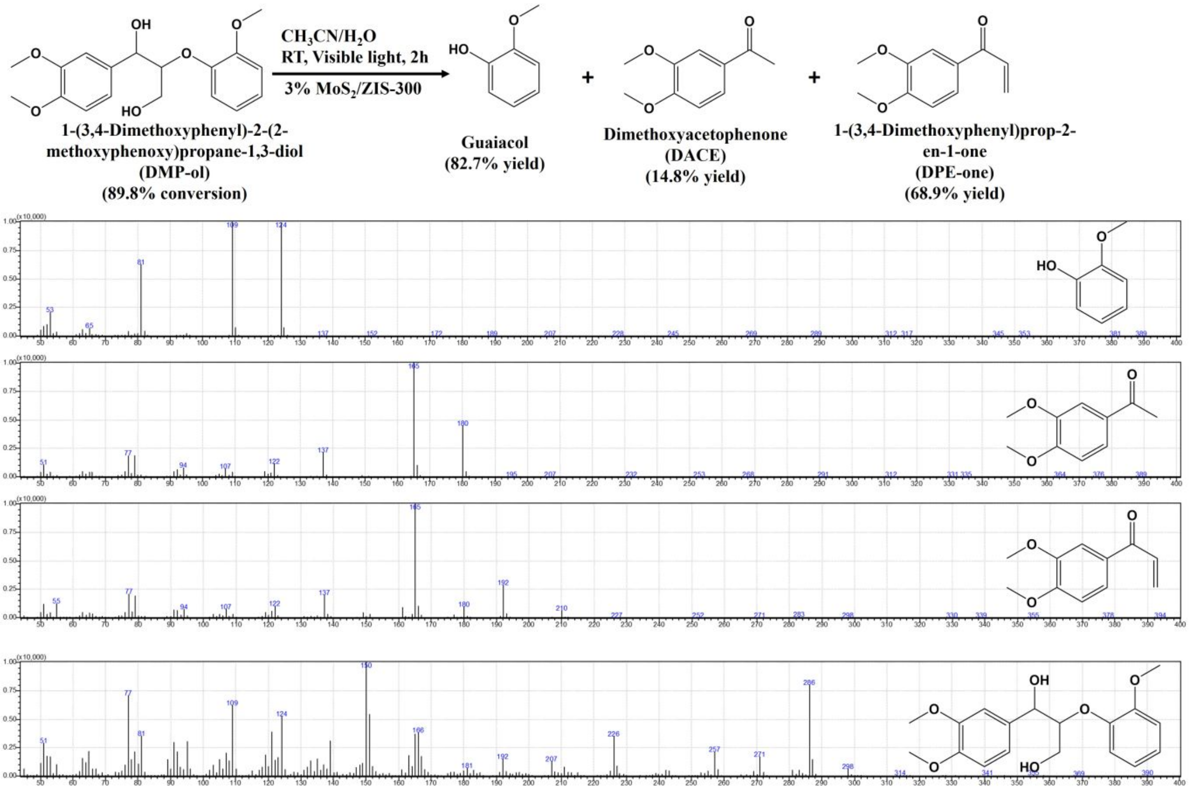

Figure S17. The detected mass spectra of the fragmented different lignin models to aromatic monomers. Reaction condition: lignin model compound is 10 mg, 3% MoS_2/ZIS-300 is 10 mg, solvent (CH_3CN/H_2O (v/v = 2/3)) is 5 mL, Ar is at 1 atm, visible light power is 0.35 W cm^{-2}, 2 h.

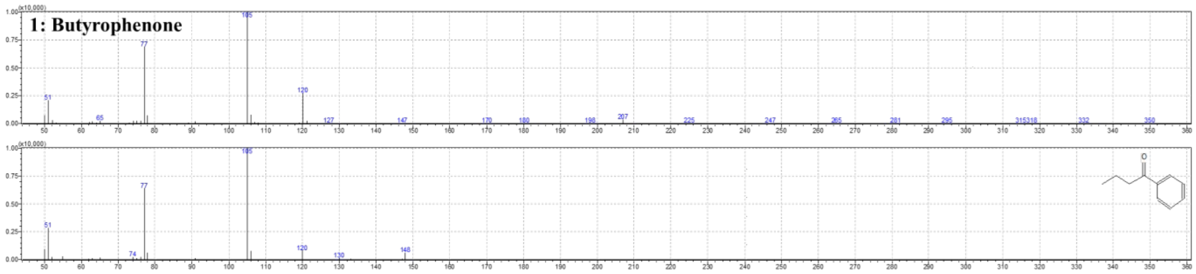
1: Butyrophenone

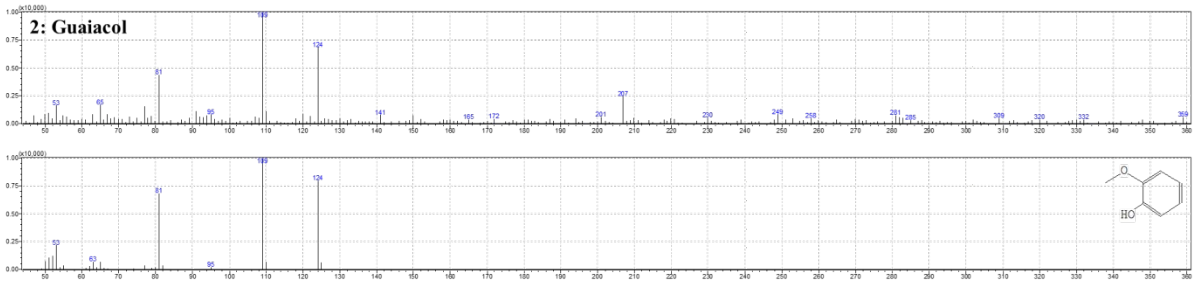
2: Guaiacol

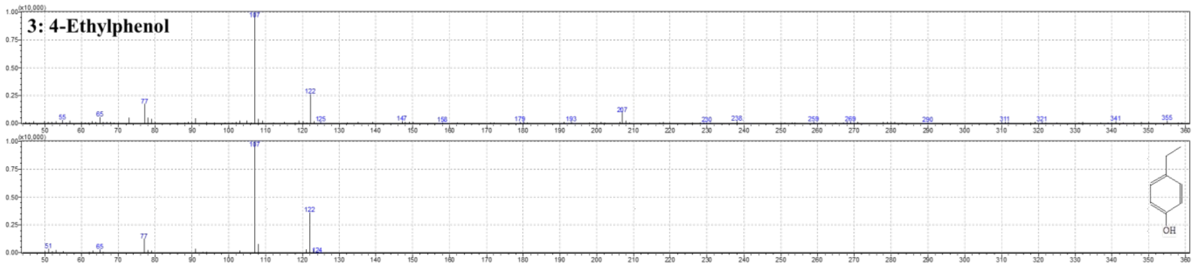
3: 4-Ethylphenol

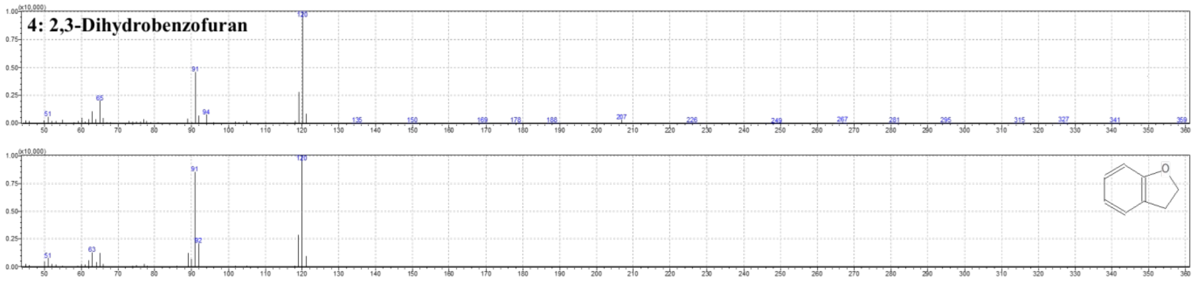
4: 2,3-Dihydrobenzofuran

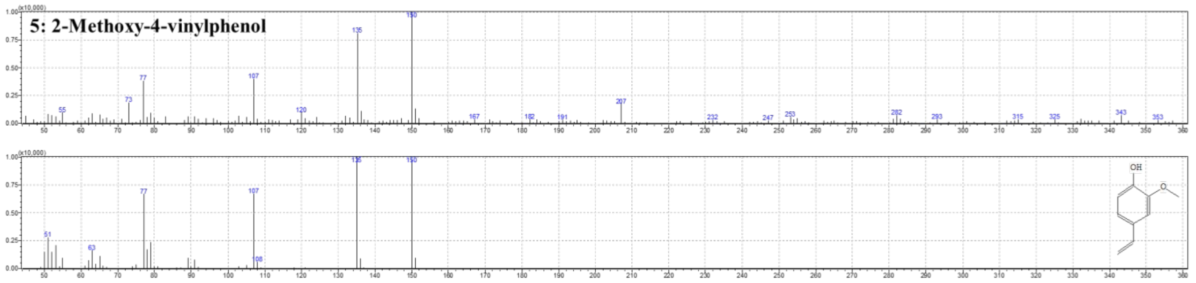
5: 2-Methoxy-4-vinylphenol

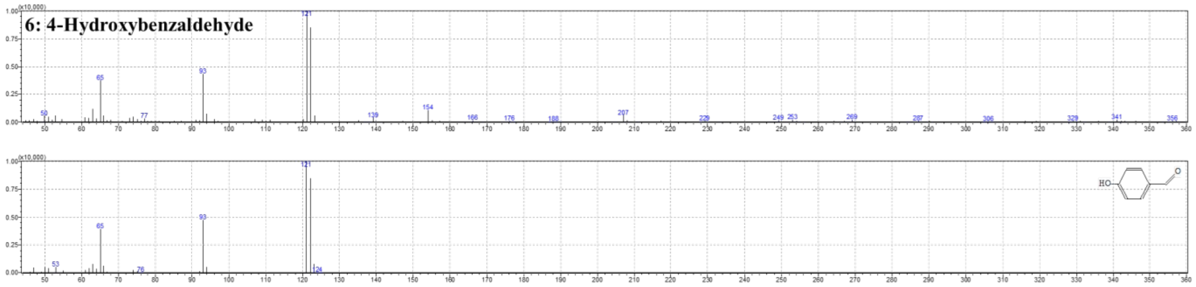
6: 4-Hydroxybenzaldehyde

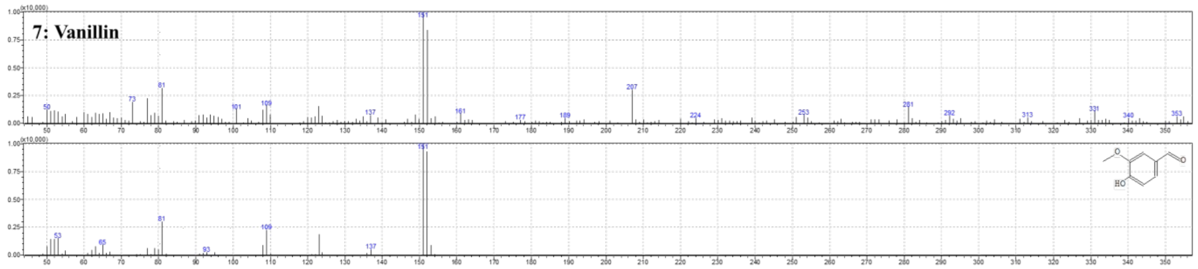
7: Vanillin

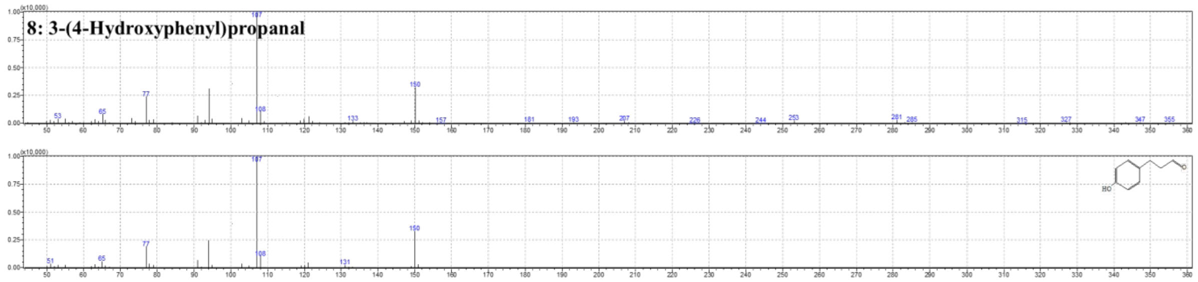
8: 3-(4-Hydroxyphenyl)propanal

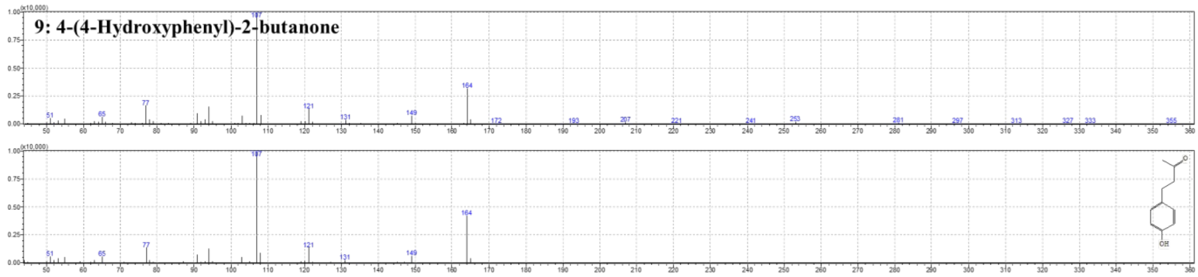
9: 4-(4-Hydroxyphenyl)-2-butanone

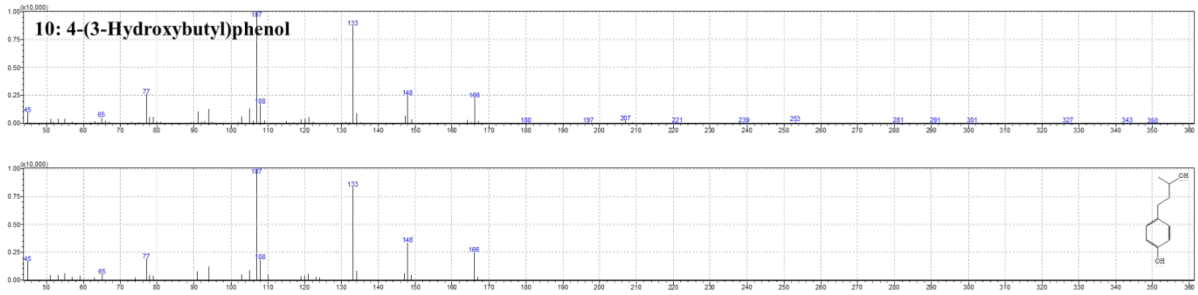
10: 4-(3-Hydroxybutyl)phenol

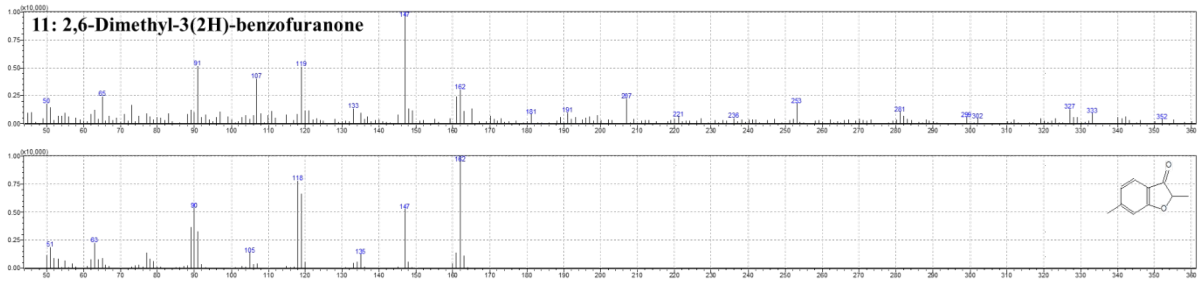
11: 2,6-Dimethyl-3(2H)-benzofuranone

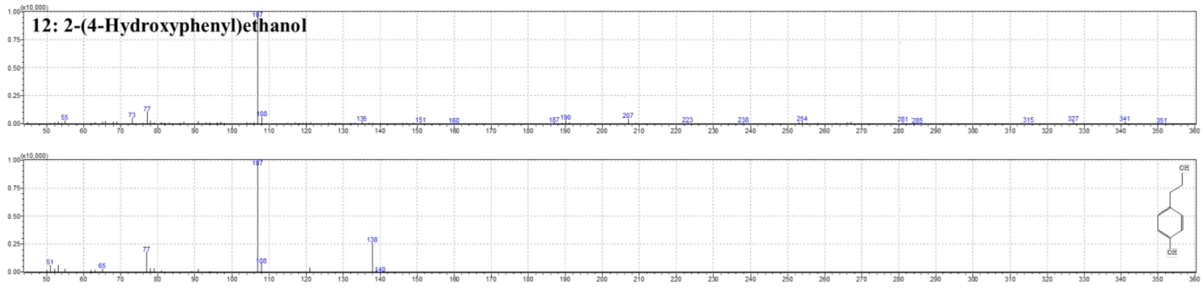
12: 2-(4-Hydroxyphenyl)ethanol

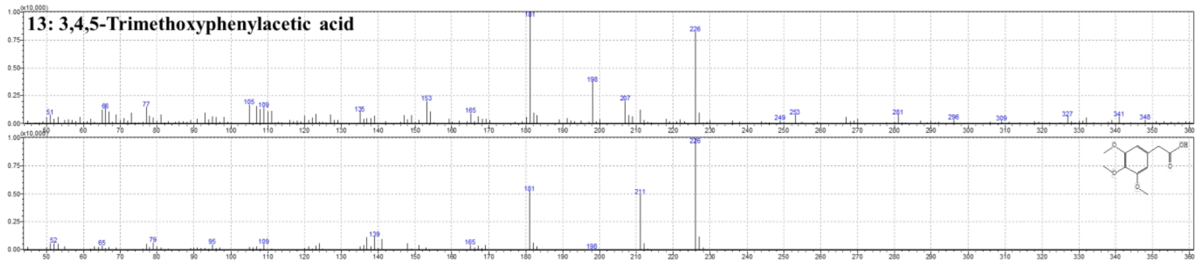

Figure S18. The detected and the standard mass spectra of different aromatic monomers from fragmentation of wood extraction lignin in 10 h of visible light irradiation. Reaction conditions: wood extraction powders are 80 mg, 3% MoS_2/ZIS-300 is 20 mg, H_2O and CH_3CN mixed solution (CH_3CN/H_2O (v/v = 2/3)) is 10 mL, Ar is at 1 atm, visible light power is 0.35 W cm^{-2}, 10 h.

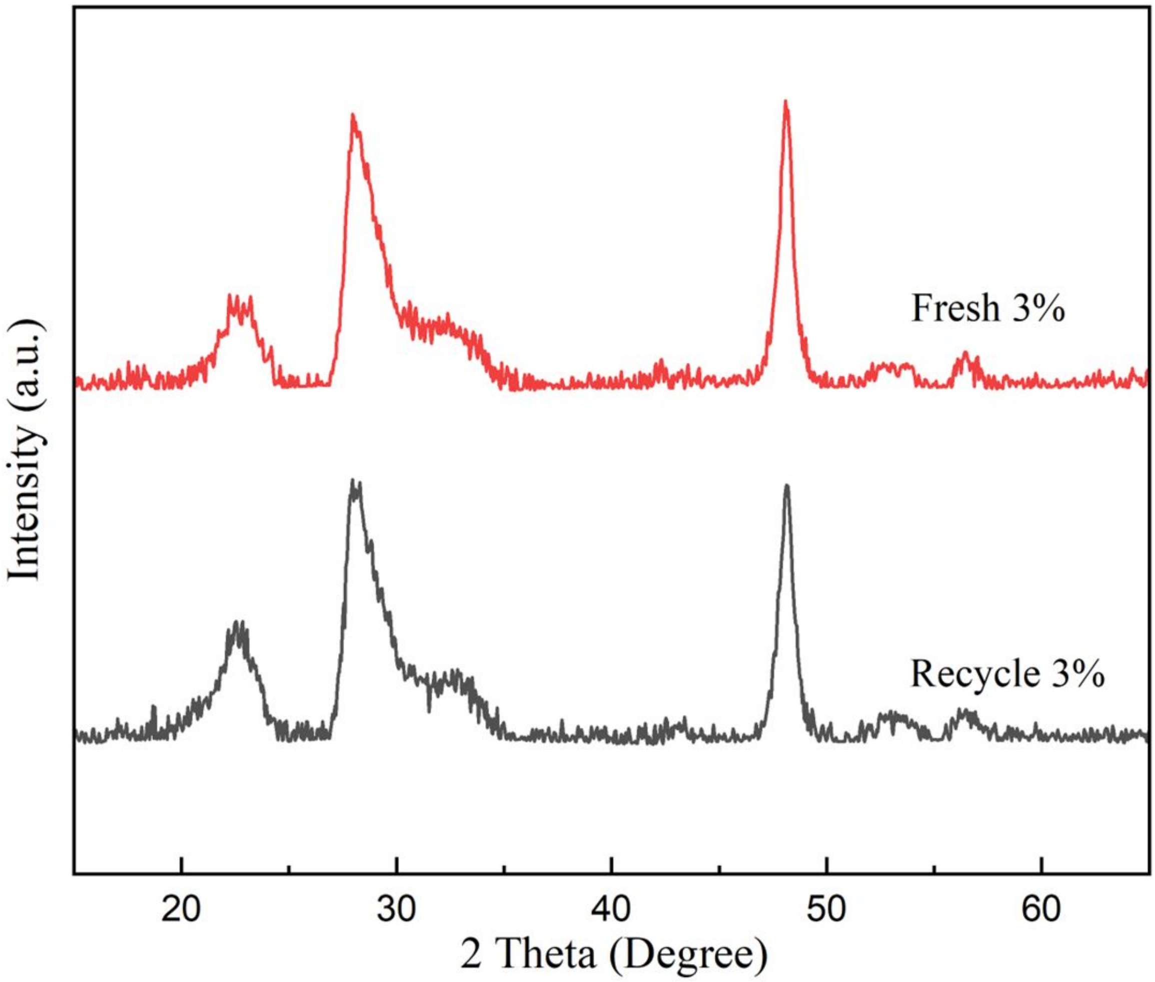

Figure S19. XRD patterns of fresh and recycled 3% MoS_2/ZIS-300.

Reference

(1) Lin, J.; Wu, X.; Xie, S.; Chen, L.; Zhang, Q.; Deng, W.; Wang, Y. Visible-Light-Driven Cleavage of C−O Linkage for Lignin Valorization to Functionalized Aromatics. *ChemSusChem* **2019**, *12* (22), 5023–5031. https://doi.org/10.1002/cssc.201902355.

(2) Jiang, Y.; Li, S.; Wang, S.; Zhang, Y.; Long, C.; Xie, J.; Fan, X.; Zhao, W.; Xu, P.; Fan, Y.; Cui, C.; Tang, Z. Enabling Specific Photocatalytic Methane Oxidation by Controlling Free Radical Type. *J. Am. Chem. Soc.* **2023**, *145* (4), 2698–2707. https://doi.org/10.1021/jacs.2c13313.

(3) Han, G.; Yan, T.; Zhang, W.; Zhang, Y. C.; Lee, D. Y.; Cao, Z.; Sun, Y. Highly Selective Photocatalytic Valorization of Lignin Model Compounds Using Ultrathin Metal/CdS. *ACS Catal.* **2019**, *9* (12), 11341–11349. https://doi.org/10.1021/acscatal.9b02842.

(4) Luo, N.; Wang, M.; Li, H.; Zhang, J.; Liu, H.; Wang, F. Photocatalytic Oxidation-Hydrogenolysis of Lignin β-O-4 Models via a Dual Light Wavelength Switching Strategy. *ACS Catal.* **2016**, *6* (11), 7716–7721. https://doi.org/10.1021/acscatal.6b02212.

(5) Perdew, J. P.; Burke, K.; Ernzerhof, M. Generalized Gradient Approximation Made Simple. *Phys. Rev. Lett.* **1996**, *77* (18), 3865.

(6) Kresse, G.; Furthmüller, J. Efficient Iterative Schemes for Ab Initio Total-Energy Calculations Using a Plane-Wave Basis Set. *Phys. Rev. B* **1996**, *54* (16), 11169.

(7) Hammer, B.; Hansen, L. B.; Nørskov, J. K. Improved Adsorption Energetics within Density-Functional Theory Using Revised Perdew-Burke-Ernzerhof Functionals. *Phys. Rev. B* **1999**, *59* (11), 7413.

(8) Grimme, S. Semiempirical GGA-type Density Functional Constructed with a Long-range Dispersion Correction. *J. Comput. Chem.* **2006**, *27* (15), 1787–1799.

(9) Yoo, H.; Lee, M. W.; Lee, S.; Lee, J.; Cho, S.; Lee, H.; Cha, H. G.; Kim, H. S. Enhancing Photocatalytic β-O-4 Bond Cleavage in Lignin Model Compounds by Silver-Exchanged Cadmium Sulfide. *ACS Catal.* **2020**, *10* (15), 8465–8475. https://doi.org/10.1021/acscatal.0c01915.

(10) Luo, N.; Wang, M.; Li, H.; Zhang, J.; Hou, T.; Chen, H.; Zhang, X.; Lu, J.; Wang, F.

Visible-Light-Driven Self-Hydrogen Transfer Hydrogenolysis of Lignin Models and Extracts into Phenolic Products. *ACS Catal.* **2017**, *7* (7), 4571–4580. https://doi.org/10.1021/acscatal.7b01043.

(11) Wu, K.; Liang, J.; Liu, S.; Huang, Y.; Cao, M.; Zeng, Q.; Li, X. Selective Photocatalytic Aerobic Oxidative Cleavage of Lignin C–O Bonds over Sodium Lignosulfonate Modified Fe3O4/TiO2. *J. Energy Chem.* **2023**, *84*, 89–100. https://doi.org/10.1016/j.jechem.2023.04.033.

(12) Dai, D.; Qiu, J.; Xia, G.; Tang, Y.; Yao, J. Defect Engineering Promoted Photocatalysis for Lignin Depolymerization: Performance and Mechanism Insight. *ACS Catal.* **2023**, *13* (22), 14987–14995. https://doi.org/10.1021/acscatal.3c03462.
